# Healthful and Unhealthful Plant-Based Diets and Chronic Obstructive Pulmonary Disease in U.S. Adults: Prospective Study

**DOI:** 10.3390/nu15030765

**Published:** 2023-02-02

**Authors:** Raphaëlle Varraso, Orianne Dumas, Fred K. Tabung, Krislyn M. Boggs, Teresa T. Fung, Frank Hu, Edward Giovannucci, Frank E. Speizer, Walter C. Willett, Carlos A. Camargo

**Affiliations:** 1 Université Paris-Saclay, UVSQ, Université Paris-Sud, Inserm, Équipe d’Épidémiologie Respiratoire Intégrative, CESP, 94807 Villejuif, France; 2 The Ohio State University College of Medicine, Comprehensive Cancer Center, Columbus, OH 43210, USA; 3 Department of Nutrition, Harvard T.H. Chan School of Public Health, Boston, MA 02115, USA; 4 Department of Emergency Medicine, Massachusetts General Hospital, Boston, MA 02114, USA; 5 Department of Nutrition, Simmons University, Boston, MA 02115, USA; 6 Department of Medicine, Channing Division of Network Medicine, Brigham and Women’s Hospital and Harvard Medical School, Boston, MA 02115, USA; 7 Department of Epidemiology, Harvard T.H. Chan School of Public Health, Boston, MA 02115, USA

**Keywords:** epidemiology, diet, healthy, feeding behavior, pulmonary disease, chronic obstructive, cohort studies

## Abstract

Background: Despite the potential protective effect of a plant-based diet against chronic obstructive pulmonary disease (COPD), it remains unknown whether intake of different types of plant foods is beneficial for COPD. Our aims were to determine whether adherence to the healthful version of a plant-based diet (healthful Plant-based Diet Index (hPDI)) is associated with a lower COPD risk, whereas adherence to the unhealthful version (unhealthful Plant-based Diet Index (uPDI)) is associated with a higher COPD risk. Methods: 46,948 men from the Health Professionals Follow-up Study, 73,592 women from the Nurses’ Health Study, and 85,515 women from the Nurses’ Health Study II who completed biennial questionnaires from 1984–2018. We derived diet scores from repeated validated food frequency questionnaires. Among 5,661,994 person-years of follow-up, we documented 2605 validated COPD cases between 1984–2018. Results: After tight control for smoking and other potential confounders, COPD risk was 46% lower among participants with the highest hPDI score compared to those with the lowest score. Conversely, COPD risk was 39% higher among participants with the highest uPDI. Further adjustment for processed meat intake led to similar results. Conclusions: These findings provide further evidence for consuming a diet that emphasizes healthful plant foods to optimize lung health.

## 1. Introduction

Chronic obstructive pulmonary disease (COPD), the third most common cause of death worldwide [1], is characterized by progressive airflow limitation and an abnormal inflammatory process in the lungs. In high-income countries, cigarette smoking is the predominant risk factor for COPD, but not all smokers develop COPD and some cases occur in never-smokers. In this context, a 2022 international expert panel report emphasized renewed efforts in COPD prevention by identifying, besides smoking, other important risk factors for COPD [2]. One such factor is diet.

Diets rich in antioxidants or in anti-inflammatory foods may modulate the effect of deleterious environmental exposures (such as smoking), or have direct benefits on lung health [3]. When making dietary recommendations it is important to investigate overall dietary patterns, rather than specific nutrients and foods, to account for the synergistic effects of the total diet on health [4]. Several studies have shown that individuals with a healthy overall diet (characterized by a high intake of fruit, whole grains, vegetables, legumes, nuts and seeds) have lower COPD risk [5,6,7]. Furthermore, more than a decade ago, we (and others since) identified processed meat intake associated with higher rates of incident COPD [8,9], a finding confirmed by a recent systematic review [10].

The term “plant-based diets” encompasses a wide range of dietary patterns that contain higher amounts of plant products and lower amounts of animal products [11]. Plant-based dietary patterns, because of their reported health benefits, are becoming increasingly popular [11]. Current evidence emphasizes the importance of a healthful plant-based dietary pattern because of its lower risk of diet-related chronic diseases, but also because of its lower environmental impact [11,12]. Indeed, a vegan eating many added sugars and refined carbohydrates (unhealthful plant-based foods) could be at greater risk for chronic diseases, compared to an omnivore consuming a variety of healthful plant-based foods and meat [11]. A differentiation of plant foods is essential and despite the potential protective effect of a plant-based diet against COPD—as reflected by the overall healthy diet [5,6,7] and a high fiber intake [13], and a low intake of processed meat [8,9,10]—it remains unknown whether overall intake of plant foods is beneficial for COPD risk.

In the context of preventing chronic diseases, there is an urgent need to develop novel strategies to prevent COPD. Improving lung health through dietary advice is particularly timely and relevant. Our aims were to determine whether adherence to the healthful version of the plant-based diet (e.g., healthful Plant-based Diet Index (hPDI)) is associated with a lower COPD risk, whereas adherence to the unhealthful version (e.g., unhealthful Plant-based Diet Index (uPDI)) is associated with a higher COPD risk.

## 2. Materials and Methods

Additional details provided in the online supplement.

### 2.1. Study Population

We analyzed data from the Health Professionals Follow-up Study (HPFS; 51,529 male US health professionals aged 40–75 years followed since 1986) [14], the Nurses’ Health Study (NHS; 121,701 female registered nurses aged 30–55 years followed since 1976) [15], and the Nurses’ Health Study II (NHSII; 116,429 female registered nurses aged 25–44 years followed since 1989) [16]. Follow-up questionnaires are sent every 2 years. Complete information on diet was collected for the first time in 1986 for HPFS, 1984 for NHS and 1991 for NHSII and were the baseline for current analyses. This investigation was approved by our local institutional review boards.

### 2.2. Dietary Intake Assessment

As previously described, we created two versions of a plant-based diet score for each food frequency questionnaire (FFQ) cycle [17], for each cohort [18]. Briefly, we summed the 18 food group scores (healthful plant food groups, unhealthful plant food groups and animal food groups) to obtain the hPDI and uPDI (Table S1, see Supplementary Materials online for details). Higher scores for the hPDI and uPDI both reflected lower animal food intake. For each cohort, we used cumulative average divided into quintiles.

### 2.3. COPD Assessment

We defined as validated COPD cases, participants who reiterated a physician diagnosis of COPD, chronic bronchitis, or emphysema, on the supplemental questionnaire sent in 1998 (HPFS and NHS), in 2000 (NHS) and in 2015 (NHSII), and who also reported a diagnostic test at diagnosis [19]. This epidemiologic definition was validated against medical records in a random sample of COPD cases in the NHS [19]. Using this validated definition, 209 cases were reported in the HPFS between 1986 and 1998, 857 cases between 1984 and 2000 in the NHS, and 583 between 1991 and 2017 in the NHSII (*n* = 1649 self-report validated COPD cases in total).

In addition, participants with COPD listed as primary cause of death (International Statistical Classification of Diseases, Ninth Revision codes; codes 491, 492 and 496) were further classified as COPD cases (*n* = 492 fatal cases in the HPFS between 1986 and 2018, *n* = 453 in the NHS between 1984 and 2018, *n* = 11 in the NHSII between 1991 and 2017). To make full-use of the lengthy study periods, we combined validated COPD cases (*n* = 1649) with fatal cases (*n* = 956) and analyzed in total 2605 cases of COPD between 1984 and 2018 (*n* = 701 in the HPFS, *n* = 1310 in the NHS and *n* = 594 in the NHSII).

### 2.4. Covariates Assessment

Covariates were obtained from the baseline questionnaire and updated every two years, when possible (Figure S1). Variables included time-varying covariates such as age, smoking status, pack years of smoking, time since quitting smoking, body mass index (BMI), physical activity, total energy intake, census tract median family income and census tract median family home value, and fixed covariates such as baseline US region and race. Smoking status was categorized as never, former, or current smoker. Pack years of smoking were calculated among ever-smokers and used as a continuous variable. Time since quitting smoking was calculated among former smokers only and used a categorical variable (quit < 1 year, 1–2 years, 2.1–5 years, 5.1–10 years, >10 years). BMI was calculated in kg/m^2^ and categorized in four categories (<20.0, 20.0–24.9, 25.0–29.9, and ≥30.0). Physical activity (walking, playing tennis, swimming, or cycling) was measured in metabolic equivalents per week and used as quintiles. Total energy intake (expressed in kilocalories per day) was estimated through the FFQ and used as a continuous variable. All residential addresses of participants at baseline and during each follow-up have been geocoded and incorporated into a Geographical Information System encompassing other spatial data layers; median income value (continuous) and median home value (continuous) have been calculated using the 1990 US Census tract [20]. US region at baseline was categorized in four classes (West, Midwest, South, Northeast). Race was categorized in two groups (white, non-white).

Asthma, also self-reported, was defined by a doctor’s diagnosis of asthma in biennial questionnaire; this definition was also validated against medical records for a random sample of asthma cases in the NHSII [16].

### 2.5. Statistical Analysis

We analyzed the association between the cumulative average of hPDI and uPDI (time-varying exposure of the average intake based on all diet questionnaires up until the time at risk) with COPD risk by using a stratified proportional Cox hazards model. The proportional hazards model was stratified according to age in months to provide finer control for age and models were adjusted for time-varying variables—smoking status, pack years of smoking and pack years of smoking squared (among ever-smokers), physical activity, total energy intake, census tract median family income and census tract median family home value [20], and fixed variables—US region at baseline and race (model 3). We further adjusted for the intake of processed meat (model 4) and total fiber intake (Table S2). After calculating cohort-specific hazard ratios, we combined hazard ratios, weighted by the inverse of their variances, by using a fixed-effects model. To test for between studies heterogeneity, we used the Cochrane Q statistic and the I2 index [21].

To address the issue of confounding vs. mediating for BMI, our main models were not adjusted for BMI, but we further adjusted for it in a sensitivity analysis (Table S3). Indeed, while low BMI and low fat-free-mass contribute to poor outcomes in COPD patients, the relationship between obesity and COPD is also increasingly recognized [22].

Smoking being the major risk factor for COPD, we further investigated the association according to smoking status. We formally tested the interaction between PDI scores and smoking, and between PDI scores and pack-years of smoking. Among former smokers, we also further adjusted for time since quitting.

We also did time lagged analyses (exclusion of cases occurring in the first 8 years of follow-up (*n* = 939), and then in the first 12 years of follow-up (*n* = 1533)).

We calculated a test for trend across the categories of PDI scores by treating the categories as an ordinal variable in a proportional hazards model. All hazard ratios (HRs) are reported with 95% confidence interval (CI). Analyses were conducted using SAS version 9.3 (Cary, NC, USA).

## 3. Results

### 3.1. Characteristics of the Population

After the exclusion of participants with missing or implausible dietary data, and those who reported a physician diagnosis of COPD or of asthma at baseline, the final baseline population included 46,948 men from HPFS, 73,592 women from NHS, and 85,515 women from NHSII (Table S4, Figure S1).

Table 1 shows participant characteristics according to quintiles of hPDI score, and Table 2 according to the quintiles of uPDI score. Compared to participants with lower hPDI scores, participants with higher scores were older and less likely to be obese; they were more likely to have quit smoking, be physically active, and report lower processed meat intake. Regarding the uPDI, participants with higher scores were younger and less likely to be former smokers and to be physically active. Correlations between the hPDI and uPDI were −0.34 in HPFS, −0.36 in NHS and −0.32 in NHSII.

In the HPFS, 49% of the men at baseline were never smokers, 42% former smokers, and only 9% current smokers; among former smokers at baseline, 57% had quit smoking at least 10 years before and only 7% had quit in the previous two years. In the NHS, 46% of the women were never smokers at baseline, 32% former smokers, and 22% current smokers; among former smokers at baseline, 48% had quit smoking at least 10 years before and only 7% had quit in the previous two years. In the NHSII in 1991, 66% were never smokers, 22% former smokers and 12% current smokers; among former smokers at baseline, 49% had quit smoking at least 10 years before, and only 12% had quit in the previous two years.

Regarding processed meat intake at baseline, 14% of the men (aged 53 years in average) and 12% of the women from the NHS (aged 50 years in average) almost never ate processed meat, whereas the corresponding figure was 56% of the women from the NHSII (aged 36 years in average).

### 3.2. Healthful Plant-Based Diet Index and COPD

Among 5,661,994 person-years of follow-up between 1984 and 2018, we documented 2605 cases of COPD (*n* = 701 in HPFS, *n* = 1310 in NHS, and *n* = 594 in NHSII).

In the pooled model, and after adjustment for several potential confounders (model 3), hPDI was inversely associated with COPD risk: pooled multivariable-adjusted HR (95% CI) for the highest compared with lowest score of hPDI was 0.54 (0.47–0.62), *p* for trend <0.001 (Table 3). Further adjustment for processed meat intake (model 4) led to similar results. In the last model, we observed a positive association between processed meat intake and COPD risk: HR (95%) were 1.15 (0.98–1.35) for <1 serving/week and 1.27 (1.07–1.52) for ≥1 servings/week, as compared to participants who reported never/almost never consuming processed meat. When we further adjusted for BMI (Table S3), or total fiber intake (Table S2), hPDI was still inversely associated with COPD risk.

When stratified according to smoking, hPDI was still inversely associated with COPD risk mostly among former and current smokers (Table S5) but the pooled interaction between hPDI and smoking was not significant (*p* = 0.95). Among ever smokers, we further investigated the interaction between hPDI and pack years of smoking, and the pooled interaction was not significant (*p* = 0.13).

When using validated COPD cases only, we still observed a strong inverse association between hPDI and COPD risk (Table S6). In the lagged analyses, firstly by excluding cases occurring in the first eight years and then in the first 12 years, we again observed similar associations (Table S7).

### 3.3. Unhealthful Plant-Based Diet Index and COPD

Conversely, but to a lesser extent, we found a positive association between uPDI and risk of COPD (Table 4). After adjustment for several potential confounders (model 3), the pooled adjusted HR (95% CI) for the highest compared with lowest score of uPDI was 1.39 (1.22–1.58), *p* for trend < 0.001 (no significant heterogeneity between studies, *p* = 0.44). Further adjustment for processed meat intake (model 4) led to similar results. In the last model, we also reported a positive association between processed meat intake and COPD risk: HR (95%) were 1.22 (1.04–1.43) for <1 serving/week and 1.41 (1.191.68) for ≥1 servings/week, as compared to participants who never/almost never ate processed meat.

When further adjusted for BMI, uPDI was still positively associated with COPD risk (Table S3). Lastly, when we further adjusted for total fiber intake (Table S2), uPDI was still positively associated with COPD risk but the association was no longer statistically significant (*p* for trend = 0.11).

When stratified according to smoking, uPDI was still positively associated with COPD risk mostly among former smokers (Table S8) but the pooled interaction between uPDI and smoking was not significant (*p* = 0.25). Among ever smokers, we further investigated the interaction between uPDI and pack years of smoking, and the pooled interaction was not significant (*p* = 0.58).

We observed similar results when using validated COPD cases only (Table S6), and in lagged analyses (Table S7).

## 4. Discussion

### 4.1. Principal Findings

Based on three prospective cohorts, with more than 206,000 U.S. men and women, we found that more adherence to a healthful version of a plant-based diet was associated with a significantly lower risk of developing COPD. By contrast, more adherence to an unhealthful version of the plant-based diet was associated, to a lesser extent, with a higher COPD risk. These associations were consistent in several sub-populations and after adjustment for many potential confounders, including detailed data on smoking history. These findings confirm the importance of differentiating plant foods, extend the relevance of healthful plant-based diet to another major chronic disease, and support the importance of diet in the pathogenesis of COPD. Furthermore, given that the two major global agendas focus on human health and environmental sustainability, choosing a healthful plant-based diet, as reflected by the hPDI, is also respecting planetary boundaries [12].

### 4.2. Comparison with Other Studies

Several dietary scores, usually based on established nutrient requirements and well-publicized dietary guidelines, have been proposed since the 2003 publication of the Mediterranean diet score [23]. To our knowledge, this is the first study to investigate the association between the quality of plant-based diet and COPD risk. Two other studies have examined overall dietary scores in relation to COPD risk: one by our group (including data from HPFS and NHS) using the Alternate Healthy Eating Index-2010 [6], and the second using a score evaluating the adherence to the Dutch dietary guidelines among participants from the Rotterdam Study [7]. Both scores evaluated overall diet quality and both studies concluded that the quality of diet was associated with COPD risk. Besides dietary scores, overall diet might be assessed using statistical techniques that summarize dietary exposure [4,24]. Using this data-driven approach, we reported, that a high intake of a Prudent pattern, characterized by a high intake of fruit, vegetables, fish, and whole-grain products, was associated with lower COPD risk in HPFS and NHS [25,26]. These findings have been confirmed by several studies since and were summarized in a recent systematic review based on eight observational studies [5]; the authors concluded that participants with healthy dietary patterns had a lower COPD risk. The current findings support the likely impact of a healthful plant-based diet in the prevention of COPD and the importance of considering the quality of plant foods, as well.

Several mechanisms may explain the observed associations. Healthful plant-based diets typically emphasize fruits, vegetables, nuts, legumes, whole grains, and other healthful plant-based foods, which contain fiber, vitamins and minerals, antioxidants, phenolic compounds, and unsaturated fatty acids. By contrast, unhealthful plant-based diets emphasize fruit juices, sugar beverages, refined grains, potatoes, and sweets, which contain refined carbohydrates and added sugars. All these foods, individually and jointly, modulate oxidative stress and systemic inflammation, two of the main pathways involved in the pathogenesis of COPD [27]. Recently, using data from a randomly selected sub-sample of women from the NHS, the authors reported that higher hPDI was associated with lower plasma concentrations of several biomarkers related to systemic inflammation (high sensitivity C-Reactive Protein, leptin), whereas higher uPDI was associated with increased concentrations of these biomarkers [28]. When we further adjusted for total fiber intake, hPDI was still inversely associated with COPD suggesting that hPDI is an important contributor to lung health, and not just a proxy for high fiber intake [13], whereas the association between uPDI and COPD was no longer significant suggesting that uPDI might be a proxy for low fiber intake. On the other hand, plant-based diets (healthful or unhealthful) also de-emphasize intake of animal products such as red and processed meats, which contain saturated fat, sodium, and nitrites, and have been positively associated with COPD risk [10,29]. In our study, when we further adjusted for processed meat intake, we still observed an inverse association between hPDI and COPD, a positive association between uPDI and COPD, and a positive association between processed meat intake and COPD, suggesting three independent associations. Our findings further encourage promotion of a diet including healthful plant foods while cutting down on unhealthful plant foods and on processed meat.

Relatively little attention has been paid to diet in the primary prevention of COPD, and how interrelationships between protective dietary factors and smoking contribute to COPD risk. Indeed, the magnitude of the association between plant-based diet on COPD may depend on other factors which influence pulmonary oxidant/antioxidant balance and inflammation, such as smoking. The stratified analyses according to smoking, which are commonly recognized as a way to address residual confounding by smoking, may pose new challenges for interpretation: are plant-based diets preferentially affecting former or current smokers, or is the effect in smokers due to residual confounding? In our study, although we reported no significant interaction between smoking and plant based dietary scores, the association between hPDI and COPD risk was mostly observed among former and current smokers, and for uPDI, among former smokers. In the two previous studies investigating the impact of the quality of diet on COPD risk [6,7], one study reported no interaction between dietary guideline score and smoking status on COPD (but the stratified analyses were not reported) [7], and the other lacked power to conduct a meaningful analysis among never smokers, and reported similar association among former and current smokers [6]. Although this issue requires further investigation, our findings support the hypothesis that both smoking and diet are important targets for effective primary prevention of COPD.

### 4.3. Strengths and Limitations of Study

The study has potential limitations. First, COPD was defined by self-report of physician-diagnosed COPD, and lung function measures were not available for these large national cohorts raising the possibility of underdiagnosis or misdiagnosis. However, our questionnaire-based definition of newly-diagnosed COPD was validated in a subset of registered nurses showing that a questionnaire-based approach to the epidemiologic study of COPD is practical among middle-aged health professionals and that a self-report of COPD from these health professionals aligns closely with documentation in the medical record [19], and has been used successfully for many years as an accepted surrogate for COPD [6,8,9,13,19,25,26,29,30,31]. Furthermore, while combining newly-diagnosed COPD with fatal cases, we acknowledge that fatal cases might represent a specific group of individuals with more severe disease features. However, our results using only validated (living) cases were similar. An important source of disease misclassification is probably misdiagnosis of asthma; however, we excluded women with asthma at baseline. Second, we acknowledge the potential role of residual confounding in all observational studies. Regarding smoking, the association between plant diet and COPD may be due, in part, to a residual confounding by cigarette smoking, which is a powerful risk factor. To minimize this possibility, multivariable models were adjusted with multiple time-varying measures of tobacco exposure; using this detailed control for smoking as time-dependent variable was able, for example, to reduce an age-adjusted relative risk of 4 for coffee consumption and lung cancer risk to a fully adjusted relative risk of 1.0 [32]. We also acknowledge that there is some misclassification of diet assessed by the food frequency questionnaire. However, the food frequency questionnaire may better capture overall dietary patterns rather than absolute levels of specific nutrients and the scores we used predict relevant biomarkers [28,33]. Finally, with regard to the sample, we note that they are all health professionals and predominantly white. The relatively homogeneity of the group actually helps with causal inferences about the relation between plant-based diet intake and COPD risk because the comparability of the high and low dietary score groups will be otherwise higher than in a more diverse population (i.e., less potential for residual confounding). However, we also recognize that the current results are not necessarily generalizable to the whole population, including other racial/ethnic populations.

## 5. Conclusions

Higher intake of a plant-based diet rich in healthful plant foods was associated with lower risk of COPD, whereas a plant-based diet that emphasizes unhealthful plant foods was associated to a lesser extent with higher risk. Besides the possible impact of diet quality on COPD risk, these findings provide further evidence for including a healthful plant-based diet on the road map to optimal lung health. Clinical and public health implications of the present study are clear: healthful plant foods may promote good lung health whereas unhealthful plant foods and processed meat may confer harm. Our findings are consistent with current recommendations to increase intake of healthful plant foods for improved health.

## Figures and Tables

**Table 1:** Baseline characteristics in participants according to quintiles of healthful Plant-based Diet Index (hPDI).

	Health Professionals Follow-Up Study (*n* = 46,948)			Nurses’ Health Study (*n* = 73,592)			Nurses’ Health Study II (*n* = 85,515)		
	Quintile 1	Quintile 3	Quintile 5	Quintile 1	Quintile 3	Quintile 5	Quintile 1	Quintile 3	Quintile 5
Age, mean (SD), years *	50.3 (8.8)	52.7 (9.3)	54.3 (9.1)	47.8 (7.0)	49.9 (7.1)	51.8 (6.8)	34.9 (4.7)	36.1 (4.6)	37.2 (4.4)
Smoking, %									
Never smokers	52	48	47	49	46	43	71	66	62
Former smokers	35	43	48	26	32	38	16	22	28
Current smokers	12	9	5	25	22	18	12	12	10
Pack-years in ever smokers, mean (SD) ^†^	19.8 (17.8)	19.3 (17.8)	17.3 (16.9)	22.0 (16.1)	20.0 (16.1)	17.9 (16.1)	12.1 (8.2)	11.0 (8.0)	10.3 (7.8)
Body mass index, mean (SD), kg/m^2^	25.1 (4.9)	25.0 (4.8)	24.5 (5.0)	25.4 (5.0)	25.0 (4.5)	24.2 (4.1)	25.1 (5.8)	24.5 (5.1)	23.5 (4.3)
Body mass index, kg/m^2^, %									
<20.0	3	3	4	8	7	9	13	13	14
20–24.9	43	43	51	49	54	58	49	53	59
25–29.9	45	46	39	27	27	24	21	21	19
≥30.0	9	8	6	16	12	9	17	13	8
Physical activity, mean (SD), METs/week ^‡^	16.1 (22.5)	20.8 (24.4)	27.0 (27.6)	10.2 (16.6)	14.0 (20.4)	19.6 (26.2)	14.7 (22.6)	20.3 (25.8)	29.0 (32.3)
Total energy intake, mean (SD), kcal/day *	2329 (622)	1944 (584)	1732 (531)	2045 (518)	1721 (497)	1486 (454)	2091 (535)	1755 (516)	1541 (471)
US region, %									
West	19	22	27	6	11	20	9	14	24
Midwest	31	27	23	18	20	19	37	33	26
South	24	28	28	8	11	12	18	21	21
Northeast	26	23	22	68	58	49	36	31	29
White race, %	92	91	90	98	98	97	96	97	96
*He l* Plant-based Diet Index	46.1 (4.2)	54.5 (3.8)	63.6 (4.8)	46.6 (4.3)	54.3 (4.0)	62.6 (4.8)	46.6 (4.4)	54.7 (3.8)	63.4 (4.9)
*Unhealthful* Plant-based Diet Index	59.4 (6.1)	54.7 (6.1)	50.5 (5.8)	60.7 (6.6)	55.0 (6.6)	49.5 (6.3)	60.1 (6.6)	54.7 (6.4)	49.8 (6.0)
Census tract median family income, mean (SD) ^§^	42,203 (18,132)	42,109 (18,952)	42,899 (19,949)	45,717 (16,317)	46,512 (17,270)	48,323 (18,792)	42,054 (14,025)	43,819 (15,178)	45,967 (16,579)
Census tract median family home value, mean (SD) ^§^	118,418 (92,853)	120,767 (99,519)	130,924 (108,775)	128,714 (84,368)	135,072 (93,969)	151,359 (108,580)	103,575 (75,998)	117,082 (88,107)	139,900 (103,939)
Food intake (servings/week), mean (SD)									
Whole grains	5.4 (7.4)	10.8 (8.6)	16.4 (11.0)	3.9 (5.8)	8.1 (6.8)	12.1 (8.3)	5.5 (6.6)	10.2 (6.9)	14.5 (8.2)
Fruits	6.1 (6.1)	10.7 (7.4)	16.9 (10.6)	5.7 (5.3)	9.7 (6.2)	14.4 (7.8)	4.5 (4.9)	8.3 (5.3)	12.7 (7.1)
Vegetables	13.6 (8.9)	20.4 (10.3)	28.8 (14.4)	13.8 (8.4)	20.3 (9.3)	27.9 (12.7)	12.6 (9.2)	20.4 (10.2)	29.3 (13.8)
Nuts	1.5 (3.0)	2.7 (3.5)	3.6 (4.0)	0.8 (1.7)	1.5 (2.1)	2.1 (2.5)	0.7 (1.3)	1.1 (1.4)	1.4 (1.7)
Legumes	2.1 (1.9)	3.0 (2.1)	4.1 (2.9)	2.1 (1.6)	2.8 (1.8)	3.5 (2.3)	1.7 (1.8)	2.6 (1.9)	3.6 (2.7)
Vegetable oils	0.9 (1.8)	1.7 (2.4)	2.5 (3.1)	1.0 (2.0)	1.9 (2.4)	3.0 (3.2)	0.9 (2.1)	2.0 (2.7)	3.1 (3.6)
Tea & Coffee	13.4 (12.9)	17.0 (13.4)	18.0 (13.6)	17.6 (12.8)	22.1 (13.5)	24.2 (14.0)	11.2 (11.9)	15.9 (12.9)	18.9 (13.4)
Fruit juices	5.7 (6.1)	5.6 (5.5)	5.1 (5.6)	5.4 (5.2)	5.2 (4.9)	4.5 (4.7)	4.6 (5.7)	4.7 (5.1)	4.4 (4.9)
Refined grains	12.6 (8.9)	10.4 (7.6)	8.7 (6.3)	14.5 (9.8)	11.6 (8.5)	9.1 (6.9)	12.4 (7.6)	10.8 (6.2)	9.9 (5.4)
Potatoes	4.7 (3.0)	3.8 (2.6)	3.2 (2.2)	4.4 (2.8)	3.5 (2.3)	2.8 (1.9)	4.7 (2.9)	3.7 (2.3)	2.8 (1.9)
Sugar sweetened beverages	4.3 (5.5)	2.2 (3.5)	1.3 (2.3)	3.6 (5.4)	1.9 (3.5)	1.1 (2.0)	5.6 (7.6)	3.0 (5.1)	1.7 (2.9)
Sweets and Desserts	12.1 (9.9)	9.8 (8.3)	7.0 (6.4)	10.7 (9.0)	8.8 (7.2)	7.1 (5.7)	10.2 (8.0)	8.5 (6.6)	6.7 (5.3)
Animal fat ^¶^	3.5 (6.0)	1.7 (3.9)	0.9 (2.4)	4.7 (7.2)	2.3 (4.7)	1.4 (3.2)	1.9 (4.2)	1.0 (2.7)	0.6 (1.8)
Dairy	14.7 (10.1)	13.5 (8.7)	12.2 (7.7)	13.5 (9.1)	13.9 (8.2)	14.1 (7.6)	15.4 (9.7)	16.1 (8.9)	16.2 (8.2)
Egg	3.0 (3.3)	2.2 (2.6)	1.5 (2.3)	2.8 (2.3)	2.5 (2.1)	2.1 (1.9)	1.6 (1.6)	1.3 (1.4)	0.9 (1.1)
Fish or Seafood	2.3 (1.9)	2.7 (2.2)	3.2 (2.6)	1.8 (1.6)	2.2 (1.7)	2.5 (2.1)	1.6 (1.5)	1.9 (1.6)	2.1 (2.0)
Meat	14.0 (5.7)	12.5 (5.3)	10.1 (5.1)	12.4 (4.9)	11.8 (4.5)	10.8 (4.4)	12.8 (4.8)	11.8 (4.5)	9.9 (4.6)
Misc. animal-based foods	3.2 (2.8)	2.8 (2.5)	2.1 (2.0)	3.3 (2.7)	3.0 (2.4)	2.5 (2.2)	3.2 (2.4)	2.8 (2.1)	2.3 (1.9)
Processed meat intake,									
Never/almost never	4	11	36	3	10	28	33	57	80
<1 serving/week	14	26	34	16	29	39	42	33	17
≥1 servings/week	83	62	30	81	61	33	25	10	3
Total fiber intake (g/week)	107.9 (36.7)	143.5 (39.3)	188.1 (54.8)	95.5 (28.3)	122.0 (29.5)	151.5 (38.2)	95.6 (30.4)	126.0 (30.1)	159.6 (42.2)

Values are means (SD) adjusted for age and total energy intake for continuous variables; percentages standardized to the age distribution of the study population for categorical variables. Values of polytomous variables may not sum to 100% due to rounding. The percentages of missing values at baseline were 0% for all variables, excepted for smoking (3.9% in Health Professionals Follow-up Study), pack-years of smoking (8.7% in Health Professionals Follow-up Study and 2.8% in Nurses’ Health Study), body mass index (4.8% in Nurses’ Health Study and 2.8% in Nurses’ Health Study II), physical activity (0.2% in Health Professionals Follow-up Study, 12.9% in Nurses’ Health Study and 0.3% in Nurses’ Health Study II), US region (0.1% in Health Professionals Follow-up Study), and census tract data (0.3% in Health Professionals Follow-up Study, 4.1% in Nurses’ Health Study and 0.3% in Nurses’ Health Study II). * Values are not age nor energy adjusted. ^†^ Number of packs smoked per day multiplied by number of years smoked, among ever smokers. ^‡^ Sum of average time per week spent in each activity multiplied by metabolic equivalent (MET) value of each activity. ^§^ Values are based on the 1990 US Census at the participant’s baseline address. ^¶^ Food items constituting the “animal fat” food group were: “Butter added to food”, and “Butter or lard used for cooking”.

**Table 2:** Baseline characteristics in participants according to quintiles of unhealthful Plant-based Diet Index (uPDI).

	Health Professionals Follow-Up Study (*n* = 46,948)			Nurses’ Health Study (*n* = 73,592)			Nurses’ Health Study II (*n* = 85,515)		
	Quintile 1	Quintile 3	Quintile 5	Quintile 1	Quintile 3	Quintile 5	Quintile 1	Quintile 3	Quintile 5
Age, mean (SD), years *	53.4 (8.9)	52.7 (9.3)	51.4 (9.5)	50.1 (6.8)	49.9 (7.1)	49.4 (7.3)	36.8 (4.5)	36.0 (4.6)	35.3 (4.8)
Smoking, %									
Never smokers	45	48	55	42	46	51	59	66	73
Former smokers	47	43	36	39	32	25	29	22	15
Current smokers	8	9	9	19	22	24	12	12	11
Pack-years in ever smokers, mean (SD) ^†^	18.9 (17.7)	19.1 (17.5)	18.1 (17.5)	18.6 (15.7)	20.1 (16.1)	21.2 (16.3)	10.7 (7.9)	11.0 (7.9)	11.5 (8.0)
Body mass index, mean (SD), kg/m^2^	25.2 (5.1)	24.9 (4.9)	24.7 (4.6)	25.1 (4.6)	24.8 (4.5)	24.7 (4.6)	24.4 (5.0)	24.4 (5.0)	24.5 (5.3)
Body mass index, kg/m^2^, %									
<20.0	3	3	3	6	8	9	11	14	17
20–24.9	42	45	48	52	54	54	54	54	51
25–29.9	45	45	43	28	26	25	22	20	19
≥30.0	10	7	6	13	12	12	13	12	13
Physical activity, mean (SD), METs/week ^‡^	24.2 (26.1)	21.1 (24.8)	17.7 (23.0)	18.9 (25.7)	14.2 (21.6)	10.2 (16.3)	26.4 (29.9)	20.8 (26.9)	16.0 (23.6)
Total energy intake, mean (SD), kcal/day *	2281 (621)	1966 (597)	1753 (555)	1959 (518)	1727 (513)	1552 (497)	2051 (526)	1772 (525)	1557 (504)
US region, %									
West	26	22	18	18	12	7	22	15	10
Midwest	26	27	29	16	20	22	25	32	39
South	26	27	27	10	10	11	18	21	22
Northeast	22	23	25	56	58	60	34	32	29
White race, %	93	91	89	98	98	97	97	97	95
*Healthful* Plant-based Diet Index	59.0 (6.4)	54.7 (6.3)	50.1 (5.8)	59.1 (6.0)	54.3 (5.9)	50.1 (5.4)	59.8 (6.2)	54.8 (6.1)	50.1 (5.4)
*Unhealthful* Plant-based Diet Index	46.6 (4.5)	54.7 (3.7)	63.0 (4.4)	46.3 (5.0)	55.1 (4.4)	64.0 (4.8)	46.0 (4.8)	54.6 (4.1)	63.8 (4.7)
Census tract median family income, mean (SD) ^§^	42,663 (18,964)	42,261 (19,128)	42,101 (18,993)	49,928 (18,747)	46,872 (17,411)	43,381 (15,698)	46,374 (16,346)	43,994 (15,235)	41,106 (13,822)
Census tract median family home value, mean (SD) ^§^	128,301 (102,569)	122,591 (100,504)	117,228 (96,963)	159,079 (106,002)	137,426 (95,607)	115,870 (81,365)	142,831 (101,658)	118,538 (89,245)	96,708 (73,164)
Food intake (servings/week), mean (SD)									
Whole grains	14.1 (10.7)	10.8 (9.4)	7.6 (7.3)	11.3 (8.7)	7.9 (7.0)	5.2 (5.4)	13.5 (8.8)	10.1 (7.4)	7.1 (5.8)
Fruits	14.6 (10.0)	11.0 (8.6)	7.9 (6.4)	13.5 (7.9)	9.6 (6.5)	6.7 (5.0)	11.7 (7.3)	8.3 (5.9)	5.6 (4.2)
Vegetables	27.9 (13.9)	20.5 (11.0)	14.3 (8.1)	28.5 (13.0)	20.2 (9.5)	13.8 (7.0)	29.6 (14.8)	20.2 (10.4)	13.2 (7.0)
Nuts	3.2 (4.3)	2.5 (3.4)	2.0 (2.8)	1.9 (2.7)	1.5 (2.1)	1.1 (1.6)	1.4 (1.8)	1.1 (1.4)	0.9 (1.1)
Legumes	3.7 (2.8)	3.0 (2.3)	2.4 (1.8)	3.5 (2.4)	2.7 (1.8)	2.2 (1.4)	3.4 (2.6)	2.6 (2.1)	1.9 (1.5)
Vegetable oils	2.5 (3.1)	1.6 (2.4)	1.0 (1.8)	3.0 (3.3)	1.9 (2.5)	1.1 (1.7)	3.3 (3.8)	1.9 (2.7)	1.0 (1.6)
Tea & Coffee	20.2 (13.9)	16.6 (13.3)	12.4 (12.1)	25.0 (13.8)	21.7 (13.5)	17.8 (12.7)	20.1 (13.2)	15.7 (12.8)	10.5 (11.5)
Fruit juices	4.6 (6.0)	5.5 (5.5)	6.4 (5.8)	4.4 (5.0)	5.2 (5.0)	5.5 (4.9)	3.9 (5.3)	4.8 (5.2)	5.0 (5.1)
Refined grains	7.8 (7.2)	10.5 (7.4)	13.1 (8.3)	8.8 (8.2)	11.8 (8.4)	14.3 (8.7)	9.1 (6.0)	11.0 (6.4)	12.6 (6.8)
Potatoes	3.1 (2.5)	3.9 (2.6)	4.7 (2.9)	2.6 (2.2)	3.6 (2.3)	4.4 (2.5)	2.7 (2.2)	3.7 (2.3)	4.6 (2.6)
Sugar sweetened beverages	0.6 (2.8)	2.4 (3.5)	4.6 (5.0)	0.4 (2.4)	2.0 (3.3)	4.1 (5.1)	0.4 (3.5)	3.1 (4.9)	6.6 (7.0)
Sweets and Desserts	5.7 (7.7)	9.8 (7.9)	13.2 (8.9)	5.7 (6.6)	9.0 (7.1)	11.9 (8.0)	5.7 (6.0)	8.6 (6.7)	11.0 (7.2)
Animal fat ^¶^	2.6 (5.4)	1.8 (4.1)	1.5 (3.5)	2.9 (5.7)	2.6 (5.2)	2.4 (4.8)	1.5 (3.6)	1.1 (2.9)	0.9 (2.4)
Dairy	14.8 (9.7)	13.5 (8.9)	12.2 (8.2)	15.9 (8.7)	13.8 (8.2)	11.7 (7.4)	18.2 (9.6)	16.0 (8.8)	13.6 (7.9)
Egg	2.8 (3.3)	2.2 (2.7)	1.9 (2.3)	3.0 (2.3)	2.4 (2.1)	1.9 (1.9)	1.5 (1.6)	1.3 (1.4)	1.0 (1.1)
Fish or Seafood	3.7 (2.6)	2.7 (2.2)	1.9 (1.7)	3.2 (2.3)	2.1 (1.6)	1.4 (1.1)	2.7 (2.0)	1.8 (1.5)	1.2 (1.1)
Meat	12.4 (5.9)	12.4 (5.4)	12.3 (5.1)	11.9 (5.0)	11.8 (4.5)	11.3 (4.2)	11.2 (5.1)	11.7 (4.7)	11.6 (4.2)
Misc. animal-based foods	3.1 (2.8)	2.8 (2.5)	2.4 (2.0)	3.4 (3.0)	3.0 (2.5)	2.6 (2.0)	3.0 (2.5)	2.8 (2.1)	2.6 (1.8)
Processed meat intake,									
Never/almost never	14	16	15	15	13	11	58	56	57
< 1 serving/week	24	25	27	29	28	29	30	32	32
≥1 servings/week	62	59	58	56	60	61	11	11	10
Total fiber intake (g/week)	167.9 (55.0)	145.0 (48.1)	123.7 (37.7)	144.3 (40.6)	121.5 (32.8)	103.9 (26.5)	151.0 (44.1)	126.0 (35.1)	104.8 (26.4)

Values are means (SD) adjusted for age and total energy intake for continuous variables; percentages standardized to the age distribution of the study population for categorical variables. Values of polytomous variables may not sum to 100% due to rounding. The percentages of missing values at baseline were 0% for all variables, excepted for smoking (3.9% in Health Professionals Follow-up Study), pack-years of smoking (8.7% in Health Professionals Follow-up Study and 2.8% in Nurses’ Health Study), body mass index (4.8% in Nurses’ Health Study and 2.8% in Nurses’ Health Study II), physical activity (0.2% in Health Professionals Follow-up Study, 12.9% in Nurses’ Health Study and 0.3% in Nurses’ Health Study II), US region (0.1% in Health Professionals Follow-up Study), and census tract data (0.3% in Health Professionals Follow-up Study, 4.1% in Nurses’ Health Study and 0.3% in Nurses’ Health Study II). * Values are not age nor energy adjusted. ^†^ Number of packs smoked per day multiplied by number of years smoked, among ever smokers. ^‡^ Sum of average time per week spent in each activity multiplied by metabolic equivalent (MET) value of each activity. ^§^ Values are based on the 1990 US Census at the participant’s baseline address. ^¶^ Food items constituting the “animal fat” food group were: “Butter added to food”, and “Butter or lard used for cooking”.

**Table 3:** Association between the healthful Plant-based Diet Index (hPDI) and the risk of newly-diagnosed chronic obstructive pulmonary disease in three cohorts of U.S. adults.

	Health Professionals Follow-Up Study (*n* = 46,948)			Nurses’ Health Study (*n* = 73,592)			Nurses’ Health Study II (*n* = 85,515)			POOLED	
	No.	Person-Years	HR (95% CI)	No.	Person-Years	HR (95% CI)	No.	Person-Years	HR (95% CI)	No.	HR (95% CI)
**Age-adjusted model 1**											
Quintile 1	181	237,482	1.00 (ref)	325	430,763	1.00 (ref)	142	457,532	1.00 (ref)	648	1.00 (ref)
Quintile 2	151	235,314	0.70 (0.56–0.86)	264	444,616	0.67 (0.57–0.79)	127	448,784	0.81 (0.64–1.03)	542	0.71 (0.63–0.80)
Quintile 3	136	234,879	0.58 (0.47–0.73)	276	437,682	0.67 (0.57–0.79)	138	459,824	0.83 (0.66–1.06)	550	0.68 (0.61–0.76)
Quintile 4	138	238,607	0.54 (0.43–0.67)	250	441,734	0.56 (0.47–0.66)	116	474,505	0.65 (0.51–0.83)	504	0.57 (0.51–0.64)
Quintile 5	95	229,662	0.36 (0.28–0.47)	195	441,329	0.38 (0.32–0.46)	71	449,283	0.38 (0.28–0.50)	361	0.38 (0.33–0.43)
*p* for trend			<0.001			<0.001			<0.001		<0.001
*p* value *											0.78
I2 ^†^											0
**Multivariable-adjusted model 2** **^‡^**											
Quintile 1	181	237,482	1.00 (ref)	325	430,763	1.00 (ref)	142	457,532	1.00 (ref)	648	1.00 (ref)
Quintile 2	151	235,314	0.76 (0.61–0.95)	264	444,616	0.72 (0.61–0.85)	127	448,784	0.87 (0.68–1.11)	542	0.77 (0.68–0.86)
Quintile 3	136	234,879	0.67 (0.53–0.83)	276	437,682	0.76 (0.65–0.89)	138	459,824	0.93 (0.74–1.18)	550	0.77 (0.69–0.87)
Quintile 4	138	238,607	0.64 (0.51–0.80)	250	441,734	0.66 (0.56–0.78)	116	474,505	0.74 (0.57–0.94)	504	0.67 (0.60–0.76)
Quintile 5	95	229,662	0.47 (0.37–0.61)	195	441,329	0.49 (0.41–0.59)	71	449,283	0.46 (0.34–0.61)	361	0.48 (0.42–0.55)
*p* for trend			<0.001			<0.001			<0.001		<0.001
*p* value *											0.87
I2 ^†^											0
**Multivariable-adjusted model 3** **^§^**											
Quintile 1	181	237,482	1.00 (ref)	325	430,763	1.00 (ref)	142	457,532	1.00 (ref)	648	1.00 (ref)
Quintile 2	151	235,314	0.78 (0.63–0.97)	264	444,616	0.76 (0.64–0.89)	127	448,784	0.90 (0.71–1.15)	542	0.79 (0.70–0.89)
Quintile 3	136	234,879	0.69 (0.54–0.86)	276	437,682	0.82 (0.69–0.97)	138	459,824	1.01 (0.79–1.30)	550	0.82 (0.73–0.92)
Quintile 4	138	238,607	0.66 (0.52–0.83)	250	441,734	0.72 (0.61–0.87)	116	474,505	0.82 (0.63–1.07)	504	0.73 (0.64–0.82)
Quintile 5	95	229,662	0.49 (0.38–0.64)	195	441,329	0.55 (0.45–0.67)	71	449,283	0.55 (0.40–0.76)	361	0.54 (0.47–0.62)
*p* for trend			<0.001			<0.001			<0.001		<0.001
*p* value *											0.59
I2 ^†^											0
**Multivariable-adjusted model 4** **^¶^**											
Quintile 1	181	237,482	1.00 (ref)	325	430,763	1.00 (ref)	142	457,532	1.00 (ref)	648	1.00 (ref)
Quintile 2	151	235,314	0.78 (0.63–0.97)	264	444,616	0.76 (0.64–0.90)	127	448,784	0.91 (0.72–1.17)	542	0.80 (0.71–0.90)
Quintile 3	136	234,879	0.68 (0.54–0.86)	276	437,682	0.83 (0.70–0.98)	138	459,824	1.03 (0.81–1.33)	550	0.83 (0.74–0.93)
Quintile 4	138	238,607	0.68 (0.54–0.86)	250	441,734	0.74 (0.62–0.88)	116	474,505	0.85 (0.65–1.11)	504	0.74 (0.66–0.84)
Quintile 5	95	229,662	0.53 (0.40–0.70)	195	441,329	0.58 (0.47–0.71)	71	449,283	0.58 (0.42–0.80)	361	0.56 (0.49–0.65)
*p* for trend			<0.001			<0.001			0.004		<0.001
*p* value *											0.64
I2 ^†^											0

A total of 701 cases occurred during 32 years of follow-up in the Health Professionals Follow-up Study (*n* = 46,948). A total of 1310 cases occurred during 34 years of follow-up in the Nurses’ Health Study (*n* = 73,592). A total of 594 cases occurred during 26 years of follow-up in the Nurses’ Health Study II (*n* = 85,515). * *p* value, test for between-studies heterogeneity. ^†^ I2, degree of heterogeneity between-studies expressed as a percent of total variance. ^‡^ Multivariable-adjusted model 2 includes age, smoking (never, former, current), pack -years of smoking (in ever smokers only; continuous), and pack-years^2^ of smoking (in ever smokers only; continuous). ^§^ Multivariable-adjusted model 3 includes model 2 variables (see above) plus physical activity (metabolic equivalent task–hours/week, in quintiles), total caloric intake (continuous), US region (West, Midwest, South, or Northeast), race (white, or non-white), census tract median family income (continuous) and census tract median family home value (continuous). ^¶^ Multivariable-adjusted model 4 includes model 3 variables (see above) plus processed meat intake (never/almost never, <1 serving/week, ≥1 servings/week).

**Table 4:** Association between the unhealthful Plant-based Diet Index (uPDI) and the risk of newly-diagnosed chronic obstructive pulmonary disease in three cohorts of U.S. adults.

	Health Professionals Follow-Up Study (*n* = 46,948)			Nurses’ Health Study (*n* = 73,592)			Nurses’ Health Study II (*n* = 85,515)			POOLED	
	No.	Person-Years	HR (95% CI)	No.	Person-Years	HR (95% CI)	No.	Person-Years	HR (95% CI)	No.	HR (95% CI)
**Age-adjusted model 1**											
Quintile 1	136	235,773	1.00 (ref)	226	444,453	1.00 (ref)	108	453,517	1.00 (ref)	470	1.00 (ref)
Quintile 2	145	235,990	1.09 (0.86–1.38)	236	446,779	1.06 (0.88–1.27)	119	454,972	1.17 (0.89–1.50)	500	1.09 (0.96–1.24)
Quintile 3	129	233,361	1.02 (0.80–1.30)	267	440,682	1.24 (1.04–1.48)	129	458,229	1.30 (1.00–1.68)	525	1.19 (1.05–1.35)
Quintile 4	161	236,895	1.27 (1.01–1.60)	278	434,053	1.34 (1.12–1.60)	107	461,976	1.12 (0.85–1.46)	546	1.27 (1.12–1.44)
Quintile 5	130	233,924	1.12 (0.88–1.43)	303	430,156	1.54 (1.29–1.83)	131	461,233	1.46 (1.13–1.88)	564	1.40 (1.24–1.58)
*p* for trend			0.14			<0.001			0.01		<0.001
*p* value *											0.10
I^2 †^											57.9
**Multivariable-adjusted model 2** **^‡^**											
Quintile 1	136	235,773	1.00 (ref)	226	444,453	1.00 (ref)	108	453,517	1.00 (ref)	470	1.00 (ref)
Quintile 2	145	235,990	1.12 (0.88–1.41)	236	446,779	1.00 (0.83–1.20)	119	454,972	1.17 (0.90–1.51)	500	1.07 (0.94–1.21)
Quintile 3	129	233,361	1.02 (0.80–1.30)	267	440,682	1.11 (0.93–1.33)	129	458,229	1.25 (0.97–1.62)	525	1.12 (0.99–1.27)
Quintile 4	161	236,895	1.26 (1.00–1.59)	278	434,053	1.18 (0.98–1.40)	107	461,976	1.04 (0.80–1.36)	546	1.17 (1.03–1.32)
Quintile 5	130	233,924	1.10 (0.86–1.40)	303	430,156	1.29 (1.08–1.53)	131	461,233	1.39 (1.08–1.81)	564	1.26 (1.11–1.42)
*p* for trend			0.23			<0.001			0.06		<0.001
*p* value *											0.57
I^2 †^											0
**Multivariable-adjusted model 3** **^§^**											
Quintile 1	136	235,773	1.00 (ref)	226	444,453	1.00 (ref)	108	453,517	1.00 (ref)	470	1.00 (ref)
Quintile 2	145	235,990	1.13 (0.89–1.43)	236	446,779	1.05 (0.87–1.26)	119	454,972	1.22 (0.94–1.60)	500	1.11 (0.98–1.26)
Quintile 3	129	233,361	1.04 (0.82–1.34)	267	440,682	1.18 (0.98–1.42)	129	458,229	1.36 (1.04–1.76)	525	1.18 (1.03–1.34)
Quintile 4	161	236,895	1.30 (1.02–1.65)	278	434,053	1.26 (1.04–1.51)	107	461,976	1.15 (0.88–1.55)	546	1.24 (1.09–1.42)
Quintile 5	130	233,924	1.12 (0.86–1.46)	303	430,156	1.36 (1.13–1.64)	131	461,233	1.58 (1.20–2.10)	564	1.39 (1.22–1.58)
*p* for trend			0.18			<0.001			0.009		<.001
*p* value *											0.44
I^2 †^											0
**Multivariable-adjusted model 4** **^¶^**											
Quintile 1	136	235,773	1.00 (ref)	226	444,453	1.00 (ref)	108	453,517	1.00 (ref)	470	1.00 (ref)
Quintile 2	145	235,990	1.13 (0.89–1.43)	236	446,779	1.03 (0.86–1.24)	119	454,972	1.21 (0.93–1.58)	500	1.10 (0.97–1.25)
Quintile 3	129	233,361	1.04 (0.81–1.33)	267	440,682	1.16 (0.97–1.39)	129	458,229	1.34 (1.03–1.74)	525	1.16 (1.02–1.32)
Quintile 4	161	236,895	1.29 (1.01–1.64)	278	434,053	1.23 (1.02–1.48)	107	461,976	1.13 (0.85–1.50)	546	1.23 (1.08–1.39)
Quintile 5	130	233,924	1.12 (0.86–1.45)	303	430,156	1.34 (1.11–1.61)	131	461,233	1.56 (1.18–2.07)	564	1.32 (1.16–1.51)
*p* for trend			0.20			<0.001			0.01		<0.001
*p* value *											0.48
I^2 †^											0

A total of 701 cases occurred during 32 years of follow-up in the Health Professionals Follow-up Study (*n* = 46,948). A total of 1310 cases occurred during 34 years of follow-up in the Nurses’ Health Study (*n* = 73,592). A total of 594 cases occurred during 26 years of follow-up in the Nurses’ Health Study II (*n* = 85,515). * *p* value, test for between-studies heterogeneity. ^†^ I2, degree of heterogeneity between-studies expressed as a percent of total variance. ^‡^ Multivariable-adjusted model 2 includes age, smoking (never, former, current), pack -years of smoking (in ever smokers only; continuous), and pack-years^2^ of smoking (in ever smokers only; continuous). ^§^ Multivariable-adjusted model 3 includes model 2 variables (see above) plus physical activity (metabolic equivalent task–hours/week, in quintiles), total caloric intake (continuous), US region (West, Midwest, South, or Northeast), race (white, or non-white), census tract median family income (continuous) and census tract median family home value (continuous). ^¶^ Multivariable-adjusted model 4 includes model 3 variables (see above) plus processed meat intake (never/almost never, <1 serving/week, ≥1 servings/week).

## Data Availability

Data cannot be shared publicly because of restriction of membership in HPFS, NHS and NHSII. Data are available from the Channing Institutional Data Access/Ethics Committee (contact via http://www.nurseshealthstudy.org/ (accessed on 15 December 2022)) for researchers who meet the criteria for access to confidential data.

## Supplementary Materials

The following supporting information can be downloaded at: https://www.mdpi.com/article/10.3390/nu15030765/s1, Supplementary Methods. Selection of studies population, assessment of variables, and statistical analyses [34,35]; Table S1. Examples of food items constituting the 18 food groups (from the 1984 Nurses’ Health Study food frequency questionnaire); Table S2. Association between the healthful Plant-based Diet Index and unhealthful Plant-based Diet Index with the risk of newly-diagnosed chronic obstructive pulmonary disease further adjusted for total fiber intake in three cohorts of U.S. adults; Table S3. Association between the healthful Plant-based Diet Index and unhealthful Plant-based Diet Index with the risk of newly-diagnosed chronic obstructive pulmonary disease further adjusted for body mass index in three cohorts of U.S. adults; Table S4. Comparison of baseline characteristics of participants included and excluded from the analyses; Table S5. Association between the healthful Plant-based Diet Index and the risk of newly-diagnosed chronic obstructive pulmonary disease in three cohorts of U.S. adults according to smoking status; Table S6. Association between the healthful Plant-based Diet Index and unhealthful Plant-based Diet Index with the risk of newly-diagnosed chronic obstructive pulmonary disease using validated definition in three cohorts of U.S. adults; Table S7. Lagged associations (8 years and 12 years) between the healthful Plant-based Diet Index and unhealthful Plant-based Diet Index with the risk of newly-diagnosed chronic obstructive pulmonary disease in three cohorts of U.S. adults; Table S8. Association between the unhealthful Plant-based Diet Index and the risk of newly-diagnosed chronic obstructive pulmonary disease in three cohorts of U.S. adults according to smoking status; Figure S1. Flowchart of participants’ selection in the Health Professional follow-up Study, Nurses’ Health Study, Nurses’ Health Study II, and their respective study designs.
