# Supplementary material for: Healthful and Unhealthful Plant-Based Diets and Chronic Obstructive Pulmonary Disease in U.S. Adults: Prospective Study
Source: Nutrients. 2023 Feb 2;15(3):765. doi: 10.3390/nu15030765 (PMC9921620; doi:10.3390/nu15030765)

Supplementary Online Content

Healthful and *unhealthful* plant-based diets and chronic obstructive pulmonary disease in U.S. adults: prospective study

Raphaëlle Varraso, Orianne Dumas, Fred K Tabung, Krislyn M Boggs, Teresa T Fung, Frank Hu, Edward Giovannucci, Frank E Speizer, Walter C Willett, Carlos A Camargo Jr

Contact Info: Raphaëlle Varraso, raphaelle.varraso@inserm.fr

INSERM U1018, CESP. 16 avenue Paul Vaillant Couturier, 94 807 Villejuif, France

Telephone: + 33 (0) 1 45 59 51 86 Fax: + 33 (0)1 45 59 51 69

Table of Contents

- **Supplementary Methods**. Selection of studies population, assessment of variables, and statistical analyses
- **Table S1.** Examples of food items constituting the 18 food groups (from the 1984 Nurses' Health Study food frequency questionnaire)
- **Table S2.** Association between the *healthful* Plant-based Diet Index and *unhealthful* Plant-based Diet Index with the risk of newly-diagnosed chronic obstructive pulmonary disease further adjusted for total fiber intake in three cohorts of U.S. adults
- **Table S3.** Association between the *healthful* Plant-based Diet Index and *unhealthful* Plant-based Diet Index with the risk of newly-diagnosed chronic obstructive pulmonary disease further adjusted for body mass index in three cohorts of U.S. adults
- **Table S4.** Comparison of baseline characteristics of participants included and excluded from the analyses
- **Table S5**. Association between the *healthful* Plant-based Diet Index and the risk of newly-diagnosed chronic obstructive pulmonary disease in three cohorts of U.S. adults according to smoking status
- **Table S6**. Association between the *healthful* Plant-based Diet Index and *unhealthful* Plant-based Diet Index with the risk of newly-diagnosed chronic obstructive pulmonary disease using validated definition in three cohorts of U.S. adults
- **Table S7**. Lagged associations (8 years and 12 years) between the *healthful* Plant-based Diet Index and *unhealthful* Plant-based Diet Index with the risk of newly-diagnosed chronic obstructive pulmonary disease in three cohorts of U.S. adults
- **Table S8**. Association between the *unhealthful* Plant-based Diet Index and the risk of newly-diagnosed chronic obstructive pulmonary disease in three cohorts of U.S. adults according to smoking status
- **Figure S1**. Flowchart of participants' selection in the Health Professional follow-up Study, Nurses' Health Study, Nurses' Health Study II, and their respective study designs

Supplementary Methods

Study population

The Health Professionals Follow-up Study began in 1986 when 51,529 male US health professionals aged 40–75 years answered a detailed mailed questionnaire that included a diet survey and items on lifestyle practice and medical history[1]. The Nurses' Health Study began in 1976, when 121,701 female registered nurses aged 30–55 years and living in 11 US states responded to a mailed health questionnaire[2]. The Nurses' Health Study II began in 1989 when 116,429 female registered nurses from 14 US states, aged 25-44 years, completed a mailed questionnaire on their medical history and lifestyle characteristics[3].

In the three cohorts, follow-up questionnaires were sent every two years thereafter to update information on smoking habits, physical activity, weight, and other risk factors and to ask about newly diagnosed medical conditions (including physician's diagnosis of emphysema or chronic bronchitis).

We used several exclusion criteria in our analysis. The first category was related to the analysis of dietary data, and the second category was related to the analysis of cohort data. Out of the 51,529 men included in the Health Professionals Follow-up Study, 51,471 answered to the main questionnaire in 1986, out of the 121,701 women included in the Nurses' Health Study, 97,381 answered to the main questionnaire in 1984, and out of the 116,429 women included in the Nurses' Health Study II, 108,214 answered to the main questionnaire in 1991. Regarding dietary data, we excluded participants with unreasonably high (>4200 kcal/day for men and >3500 kcal/day for women) or low intakes (<800 kcal/day for men and <500 kcal/day for women) to take care of outliers, as well as those who had left more than 70 items blank (n=1,596 in the Health Professionals Follow-up Study, 15,688 in the Nurses' Health Study, and 12,982 in the Nurses' Health Study II). We also excluded men and women who reported a diagnosis of asthma at baseline (n=2,502 in the Health Professionals Follow-up Study, 4,294 in the Nurses' Health Study, and 6,150 in the Nurses' Health Study II), a diagnosis of COPD at baseline confirmed in the supplemental COPD form (n=28 in the Health Professionals Follow-up Study, 188 in the Nurses' Health Study, and 4 in the Nurses' Health Study II), or any report of COPD from baseline or during the follow-up not confirmed in the supplemental COPD form (n=397 in the Health Professionals Follow-up Study, 3,619 in the Nurses' Health Study, and 3,563 in the Nurses' Health Study II).

Comparison between participants included and those excluded from the analyses are presented in **Table S2** in the Supplement.

Diet assessment

Dietary intake information was collected by a food frequency questionnaire (FFQ) designed to assess average food intake over the previous 12 months[4]. Standard portion sizes were listed with each food. For each food item, participants indicated their average frequency of consumption over the previous year in terms of the specified serving size by checking one of nine frequency categories ranging from "never or less than once per month" (e.g., never/almost never) to "at least six times/day." The selected frequency category for each food item was converted to a daily intake. For example, a response of "one serving/week" was converted to 0.14 servings/day.

Dietary intake information was collected by using semi-quantitative validated food frequency questionnaires (FFQ) designed to assess average food intake over the previous 12 months in 1986, 1990, 1994, 1998, 2002, 2006, 2010 and 2014 in the Health Professionals Follow-up Study; in 1984, 1986, 1990, 1994, 1998, 2002, 2006, 2010 and 2014 in the Nurses' Health Study; and in 1991, 1995, 1999, 2003, 2007, 2011 and 2015 in the Nurses' Health Study II.

As previously described, two versions of a plant-based diet score for each food frequency questionnaire cycle have been created, for each cohort[5.] Briefly, 18 food groups based on nutrient and culinary similarities within the larger categories of healthful plant foods (whole grains, fruits, vegetables, nuts, legumes, vegetable oils, tea and coffee), unhealthful plant foods (fruit juices, refined grains, potatoes, sugar sweetened beverages, sweets and desserts), and animal foods (animal fat, dairy, egg, fish or seafood, meat, other animal-based foods) were selected (**Table S1**). Each food group was ranked into quintiles and given positive or reverse scores. With positive scores, participants above the highest quintile of a food group received a score of 5, following on through to participants below the lowest quintile who received a score of 1. With reverse scores, this pattern of scoring was inversed. For creating the *healthful* Plant-based Diet Index (hPDI), positive scores were given to healthful plant food groups and reverse scores to unhealthful plant food groups and animal food groups, and for the *unhealthful* Plant-based Diet Index (uPDI), positive scores were given to unhealthful plant food groups and reverse scores to healthful plant food groups and animal food groups. The 18 food group scores were summed to obtain the indices and higher intake of both indices reflected lower animal food intake.

To reduce measurement errors and to represent long term dietary intake, we calculated the cumulative average of hPDI and uPDI and used it as a time dependent variable. The cumulative average incorporated repeated measures of diet. For example, in the Nurses' Health Study II, we used the 1991 hPDI to predict newly diagnosed COPD in 1991-95, an average of the 1991 and 1995 hPDI to predict COPD in 1995-99, and the average of the 1991, 1995 and 1999 to predict COPD from 1999 to 2003, and so on.

Nutrient intakes were computed by multiplying the frequency response by the nutrient content of the specified portion sizes. Food composition values were obtained from the Harvard University Food Composition Database, which was derived from US Department of Agriculture sources and supplemented with information from the manufacturer. In the US Department of Agriculture database, dietary fiber was determined by enzymatic-gravimetric methods 985.29 and 991.43 of the Association of Official Analytical Chemists[6]. We considered total fiber adjusted for total energy by using the residual method[7] as a continuous variable.

Cumulative average of processed meat consumption (sausages, salami, bacon and hot dogs) was divided into three categories: never/almost never, <1 serving/week, and ≥1 servings/week[8].

COPD assessment

Supplemental COPD questionnaires were sent to every participant who reported a physician's diagnosis of emphysema or chronic bronchitis (on biennial questionnaires) in 1998 (HPFS and NHS), in 2000 (NHS) and in 2015 (NHSII). The specific questionnaire included, among other data, information confirming a physician's diagnosis of emphysema, chronic bronchitis, or COPD, as well as the dates of symptom onset and diagnosis and the tests used to confirm the diagnosis or symptoms (consistent with a diagnosis of chronic bronchitis). Self-reported COPD cases were defined as participants who: 1) previously reported a physician-diagnosed of chronic bronchitis or emphysema on the original main questionnaires, and 2) answered positively to physician-diagnosed chronic bronchitis, or physician-diagnosed emphysema, or physician-diagnosed COPD on supplemental COPD questionnaires, and 3) reported of a diagnostic test at diagnosis (pulmonary function testing, chest radiograph, or chest computed tomography). This epidemiologic definition was validated against medical records in a random sample of COPD cases in the NHS (84% were confirmed), showing that a questionnaire-based approach to the epidemiologic study of COPD is practical among middle-aged health professionals and that a self-report of COPD from these health professionals is a valid marker of medical record evidence of COPD[9]. Using this validated definition, 209 cases were reported in the HPFS between 1986 and 1998, 857 cases between 1984 and 2000 in the NHS, and 583 between 1991 and 2017 in the NHSII (n=1,649 self-report validated COPD cases in total).

In addition, participants with COPD listed as primary cause of death (International Statistical Classification of Diseases, Ninth Revision codes; codes 491, 492 and 496) were further classified as COPD cases (n=492 fatal cases in the HPFS between 1986 and 2018, n=453 in the NHS between 1984 and 2018, n=11 in the NHSII between 1991 and 2017). To make full-use of the lengthy study periods, we combined validated COPD cases (n=1,649) with fatal cases (n=956) and analyzed in total 2,605 cases of COPD between 1984 and 2018 (n=701 in the HPFS, n=1,310 in the NHS and n=594 in the NHSII).

Statistical analyses

The percentages of missing values at baseline were 0% for all variables, excepted for smoking (3.9% in the Health Professionals Follow-up Study), pack-years of smoking (8.7% in the Health Professionals Follow-up Study and 2.8% in the Nurses' Health Study), body mass index (4.8% in the Nurses' Health Study and 2.8% in the Nurses' Health Study II), physical activity (0.2% in the Health Professionals Follow-up Study, 12.9% in the Nurses' Health Study and 0.3% in the Nurses' Health Study II), US region (0.1% in the Health Professionals Follow-up Study), and census tract data (0.3% in the Health Professionals Follow-up Study, 4.1% in the Nurses' Health Study and 0.3% in the Nurses' Health Study II). To minimize missing continuous covariates, e.g., pack years of smoking, total energy intake, census tract median family income and census tract median family home value, we replaced missing data on these four covariates with the last valid values and we created a dummy variable when making categories for these continuous covariates. Similarly, we used missing indicator variables to include participants with missing categorical variables, including smoking status, time since quitting smoking, physical activity, US region and race.

The variables included in the model are based both on the prior biological knowledge and confounders usually reported in the literature.

We tested for between studies heterogeneity by using the Cochrane Q statistic, which gives information about the presence versus the absence of heterogeneity, and we also provided the I2 index to quantify the degree of heterogeneity between studies, expressed as a percentage of total variance (10).

To avoid potential for preclinical COPD leading to reverse causation, we did time lagged analyses, by omitting the cases from the initial 8 years of follow-up (n=939), and further examined the long latency of COPD, by omitting the cases from the initial 12 years of follow-up (n=1,533).

eReferences

1. Colditz GA, Rimm EB, Giovannucci E, Stampfer MJ, Rosner B, Willett WC. A prospective study of parental history of myocardial infarction and coronary artery disease in men. *Am J Cardiol* 1991;67:933-938.
2. Colditz GA, Martin P, Stampfer MJ, et al. Validation of questionnaire information on risk factors and disease outcomes in a prospective cohort study of women. *Am J Epidemiol* 1986;123:894-900.
3. Camargo CA, Weiss ST, Zhang S, Willett WC, Speizer FE. Prospective study of body mass index, weight change, and risk of adult-onset asthma in women. *Arch Intern Med* 1999;159:2582-2588.
4. Willett WC, Sampson L, Stampfer MJ, et al. Reproducibility and validity of a semiquantitative food frequency questionnaire. *Am J Epidemiol* 1985;122:51-65.
5. Satija A, Bhupathiraju SN, Spiegelman D, et al. Healthful and unhealthful plant-based diets and the risk of coronary heart disease in U.S. Adults. *J Am Coll Cardiol* 2017;70:411-422.

6. Association of Official Analytical Chemists. Official Methods of Analysis. 16th ed. Gaithersburg, MD: Association of Official Analytical Chemists, International; 1995
7. Willett W, Stampfer MJ. Total energy intake: implications for epidemiologic analyses. *Am J Epidemiol* 1986;124:17–27.
8. Varraso R, Dumas O, Boggs KM, Willett WC, Speizer FE, Camargo CA. Processed meat intake and risk of chronic obstructive pulmonary disease among middle-aged women. *EClinicalMedicine* 2019;14:88-95.
9. Barr RG, Herbstman J, Speizer FE, Camargo CA. Validation of self-reported chronic obstructive pulmonary disease in a cohort study of nurses. Am J Epidemiol 2002;155:965-971.
10. Huedo-Medina TB, S√†nchez-Meca J, Mar√¨n-Mart√¨nez F, Botella J. Assessing heterogeneity in meta-analysis: Q statistic or I2 index? Psychol Methods 2006;11:193-206.

Table S1. Examples of food items constituting the 18 food groups (from the 1984 Nurses' Health Study food frequency questionnaire)

		healthful Plant-based Diet Index (hPDI)	*unhealthful* Plant-based Diet Index (uPDI)
**PLANT FOOD GROUPS**			
Healthful			
Whole grains	Whole grain breakfast cereal, other cooked breakfast cereal, cooked oatmeal, dark bread, brown rice, other grains, bran, wheat germ, popcorn	Positive scores	Reverse scores
Fruits	Raisins or grapes, prunes, bananas, cantaloupe, watermelon, fresh apples or pears, oranges, grapefruit, strawberries, blueberries, peaches or apricots or plums	Positive scores	Reverse scores
Vegetables	Tomatoes, tomato juice, tomato sauce, broccoli, cabbage, cauliflower, Brussels sprouts, carrots, mixed vegetables, yellow or winter squash, eggplant or zucchini, yams or sweet potatoes, spinach cooked, spinach raw, kale or mustard or chard greens, iceberg or head lettuce, romaine or leaf lettuce, celery, mushrooms, beets, alfalfa sprouts, garlic, corn	Positive scores	Reverse scores
Nuts	Nuts, peanut butter	Positive scores	Reverse scores
Legumes	String beans, tofu or soybeans, beans or lentils, peas or lima beans	Positive scores	Reverse scores
Vegetable oils	Oil-based salad dressing, vegetable oil used for cooking	Positive scores	Reverse scores
Tea & Coffee	Tea, coffee, decaffeinated coffee	Positive scores	Reverse scores
Unhealthful			
Fruit juices	Apple cider (non-alcoholic) or juice, orange juice, grapefruit juice, other fruit juice	Reverse scores	Positive scores
Refined grains	Refined grain breakfast cereal, white bread, English muffins or bagels or rolls, muffins or biscuits, white rice, pancakes or waffles, crackers, pasta	Reverse scores	Positive scores
Potatoes	French fries, baked or mashed potatoes, potato or corn chips	Reverse scores	Positive scores
Sugar sweetened beverages	Colas with caffeine & sugar, colas without caffeine but with sugar, other carbonated beverages with sugar, non-carbonated fruit drinks with sugar	Reverse scores	Positive scores
Sweets and Desserts	Chocolates, candy bars, candy without chocolate, cookies (home-baked & ready-made), brownies, doughnuts, cake (home-baked & ready-made), sweet roll (home-baked & ready-made), pie (home-baked & ready-made), jams or jellies or preserves or syrup or honey	Reverse scores	Positive scores
**ANIMAL FOOD GROUPS**			
Animal fat	Butter added-food, butter or lard used for cooking	Reverse scores	Reverse scores
Dairy	Skim low fat milk, whole milk, cream, sour cream, sherbet, ice cream, yogurt, cottage or ricotta cheese, cream cheese, other cheese	Reverse scores	Reverse scores
Egg	Eggs	Reverse scores	Reverse scores

Fish or Seafood	Canned tuna, dark meat fish, other fish, shrimp or lobster or scallops	Reverse scores	Reverse scores
Meat	Chicken or turkey with skin, chicken or turkey without skin, bacon, hot dogs, processed meats, liver, hamburger, beef or pork or lamb mixed dish, beef or pork or lamb main dish	Reverse scores	Reverse scores
Misc. animal-based foods	Pizza, chowder or cream soup, mayonnaise or other creamy salad dressing	Reverse scores	Reverse scores

Food groups were ranked into quintiles and given positive or reverse scores. With positive scores, participants above the highest quintile of a food group received a score of 5, following on through-participants below the lowest quintile who received a score of 1. With reverse scores, this pattern of scoring was inversed.

For creating hPDI, positive scores were given to healthful plant food groups and reverse scores to unhealthful plant food groups and animal food groups.

Finally, for uPDI, positive scores were given to unhealthful plant food groups and reverse scores to healthful plant food groups and animal food groups.

The 18 food group scores were summed to obtain the indices. Higher intake of all indices reflected lower animal food intake.

Table S2. Association between the *healthful* Plant-based Diet Index and *unhealthful* Plant-based Diet Index with the risk of newly-diagnosed chronic obstructive pulmonary disease further adjusted for total fiber intake in three cohorts of U.S. adults

	Health Professionals Follow-up Study (n=46,948)			Nurses' Health Study (n=73,592)			Nurses' Health Study II (n=85,515)			POOLED	
	No.	Person-Years	HR (95% CI)	No.	Person-Years	HR (95% CI)	No.	Person-Years	HR (95% CI)	No.	HR (95% CI)
***healthful* Plant-based Diet Index**											
Multivariable-adjusted model*											
Quintile 1	181	237,482	1.00 (ref)	325	430,763	1.00 (ref)	142	457,532	1.00 (ref)	648	1.00 (ref)
Quintile 2	151	235,314	0.85 (0.698-1.07)	264	444,616	**0.78 (0.66-0.93)**	127	448,784	0.91 (0.70-1.17)	542	**0.83 (0.74-0.94)**
Quintile 3	136	234,879	0.79 (0.61-1.01)	276	437,682	0.87 (0.72-1.04)	138	459,824	1.02 (0.78-1.34)	550	**0.88 (0.77-1.00)**
Quintile 4	138	238,607	0.83 (0.64-1.07)	250	441,734	**0.78 (0.64-0.95)**	116	474,505	0.84 (0.62-1.14)	504	**0.81 (0.70-0.93)**
Quintile 5	95	229,662	**0.70 (0.51-0.97)**	195	441,329	**0.62 (0.49-0.78)**	71	449,283	**0.57 (0.39-0.84)**	361	**0.63 (0.53-0.75)**
P for trend			**.048**			**<.001**			**.02**		**<.0001**
P value†											.87
I^2‡											0.0
***unhealthful* Plant-based Diet Index**											
Multivariable-adjusted model*											
Quintile 1	136	235,773	1.00 (ref)	226	444,453	1.00 (ref)	108	453,517	1.00 (ref)	470	1.00 (ref)
Quintile 2	145	235,990	1.03 (0.81-1.31)	236	446,779	0.99 (0.82-1.19)	119	454,972	1.18 (0.90-1.55)	500	1.04 (0.92-1.19)
Quintile 3	129	233,361	0.89 (0.69-1.15)	267	440,682	1.08 (0.90-1.31)	129	458,229	1.28 (0.97-1.69)	525	1.07 (0.94-1.22)
Quintile 4	161	236,895	1.04 (0.81-1.33)	278	434,053	1.12 (0.92-1.36)	107	461,976	1.07 (0.79-1.45)	546	1.09 (0.95-1.25)
Quintile 5	130	233,924	0.83 (0.63-1.09)	303	430,156	1.18 (0.96-1.44)	131	461,233	**1.45 (1.06-1.98)**	564	1.12 (0.97-1.29)
P for trend			.28			**.05**			.09		.11
P value†											.07
I^2‡											62.5

A total of 701 cases occurred during 32 years of follow-up in the Health Professionals Follow-up Study (n=46,948). A total of 1,310 cases occurred during 34 years of follow-up in the Nurses' Health Study (n=73,592). A total of 594 cases occurred during 26 years of follow-up in the Nurses' Health Study II(n=85,515).

* Multivariable-adjusted model includes age, smoking (never, former, current), pack -years of smoking (in ever smokers only; continuous), pack-years^2 of smoking (in ever smokers only; continuous), physical activity (metabolic equivalent task–hours/week, in quintiles), total caloric intake (continuous), US region (West, Midwest, South, or Northeast), race (white, or non-white), census tract median family income (continuous), census tract median family home value (continuous) and total fiber intake (continuous).

† P value, test for between-studies heterogeneity

‡ I^2, degree of heterogeneity between-studies expressed as a percent of total variance.

Table S3. Association between the *healthful* Plant-based Diet Index and *unhealthful* Plant-based Diet Index with the risk of newly-diagnosed chronic obstructive pulmonary disease further adjusted for body mass index in three cohorts of U.S. adults

	Health Professionals Follow-up Study (n=46,948)			Nurses' Health Study (n=73,592)			Nurses' Health Study II (n=85,515)			POOLED	
	No.	Person-Years	HR (95% CI)	No.	Person-Years	HR (95% CI)	No.	Person-Years	HR (95% CI)	No.	HR (95% CI)
***healthful* Plant-based Diet Index**											
Multivariable-adjusted model*											
Quintile 1	181	237,482	1.00 (ref)	325	430,763	1.00 (ref)	142	457,532	1.00 (ref)	648	1.00 (ref)
Quintile 2	151	235,314	**0.78 (0.62-0.97)**	264	444,616	**0.77 (0.65-0.90)**	127	448,784	0.91 (0.71-1.16)	542	**0.80 (0.71-0.90)**
Quintile 3	136	234,879	**0.68 (0.54-0.86)**	276	437,682	**0.84 (0.70-0.99)**	138	459,824	1.02 (0.79-1.30)	550	**0.83 (0.74-0.93)**
Quintile 4	138	238,607	**0.66 (0.52-0.84)**	250	441,734	**0.73 (0.61-0.88)**	116	474,505	0.83 (0.64-1.08)	504	**0.73 (0.65-0.83)**
Quintile 5	95	229,662	**0.49 (0.38-0.64)**	195	441,329	**0.56 (0.46-0.68)**	71	449,283	**0.56 (0.41-0.77)**	361	**0.54 (0.47-0.62)**
P for trend			**<.001**			**<.001**			**.001**		**<.001**
P value†											.50
I^2‡											0.0
***unhealthful* Plant-based Diet Index**											
Multivariable-adjusted model*											
Quintile 1	136	235,773	1.00 (ref)	226	444,453	1.00 (ref)	108	453,517	1.00 (ref)	470	1.00 (ref)
Quintile 2	145	235,990	1.13 (0.89-1.43)	236	446,779	1.01 (0.84-1.21)	119	454,972	1.22 (0.94-1.59)	500	1.09 (0.96-1.24)
Quintile 3	129	233,361	1.04 (0.81-1.34)	267	440,682	1.13 (0.94-1.35)	129	458,229	**1.35 (1.03-1.76)**	525	**1.15 (1.01-1.31)**
Quintile 4	161	236,895	**1.28 (1.00-1.62)**	278	434,053	1.19 (0.99-1.43)	107	461,976	1.15 (0.87-1.53)	546	**1.21 (1.06-1.37)**
Quintile 5	130	233,924	1.10 (0.85-1.43)	303	430,156	**1.25 (1.04-1.51)**	131	461,233	**1.60 (1.20-2.12)**	564	**1.28 (1.12-1.46)**
P for trend			.25			**.004**			**.008**		**<.001**
P value†											.46
I^2‡											0.0

A total of 701 cases occurred during 32 years of follow-up in the Health Professionals Follow-up Study (n=46,948). A total of 1,310 cases occurred during 34 years of follow-up in the Nurses' Health Study (n=73,592). A total of 594 cases occurred during 26 years of follow-up in the Nurses' Health Study II(n=85,515).

* Multivariable-adjusted model includes age, smoking (never, former, current), pack -years of smoking (in ever smokers only; continuous), pack-years^2 of smoking (in ever smokers only; continuous), physical activity (metabolic equivalent task–hours/week, in quintiles), total caloric intake (continuous), US region (West, Midwest, South, or Northeast), race (white, or non-white), census tract median family income (continuous), census tract median family home value (continuous) and body mass index (<20 kg/m^2, 20-24.9 kg/m^2, 25-29.9 kg/m^2, or ≥30.0 kg/m^2)

† P value, test for between-studies heterogeneity

‡ I^2, degree of heterogeneity between-studies expressed as a percent of total variance.

Table S4. Comparison of baseline characteristics of participants included and excluded from the analyses

	Health Professionals Follow to up Study			Nurses' Health Study			Nurses' Health Study II		
	Included (n=46,948)	Excluded (n=4,581)	P‡	Included (n=73,592)	Excluded (n=48,109)	P‡	Included (n=85,515)	Excluded (n=30,914)	P‡
Age, mean (SD), years	54.2 (10.1)	55.2 (10.7)	<.001	50.5 (7.2)	51.2 (7.2)	<.001	36.1 (4.7)	36.2 (4.7)	.001
Smoking									
Never smokers	44	44	.77	44	39	<.001	66	62	<.001
Former smokers	42	43		32	25		22	23	
Current smokers	10	10		24	24		12	16	
Missing	4	4		0	13		0	0	
Pack-years in ever smokers, mean (SD)*	22.9 (17.8)	24.5 (19.2)	<.001	21.7 (17.8)	24.1 (19.3)	<.001	11.5 (8.4)	12.7 (9.6)	<.001
Body mass index, mean (SD), kg/m^2	24.9 (5.1)	24.9 (5.4)	.48	25.0 (4.7)	25.6 (5.1)	<.001	24.5 (5.2)	25.6 (6.0)	<.001
Body mass index, kg/m^2									
< 25.0	48	48	.03	57	21	<.001	65	42	<.001
25-29.9	44	43		25	11		20	16	
≥ 30.0	8	9		12	6		12	13	
Missing	0	0		5	61		3	29	
Physical activity, mean (SD), METs/week†	20.4 (24.6)	20.1 (26.4)	.40	14.2 (21.1)	13.6 (20.6)	<.001	21.0 (27.4)	20.9 (27.9)	.59
US region									
West	22	24	<.001	12	13	<.001	15	16	<.001
Midwest	27	26		19	17		32	29	
South	27	29		11	12		21	24	
Northeast	23	21		58	59		32	31	
White race	91	89	<.001	98	95	<.001	96	93	<.001

Values are percentages unless stated otherwise. SD: standard deviation.

* Number of packs smoked per day multiplied by number of years smoked, among ever smokers.

† Sum of average time per week spent in each activity multiplied by metabolic equivalent (MET) value of each activity.

‡ P for heterogeneity were assessed using Chi square test for dichotomous variables and linear regression for continuous variables.

Table S5. Association between the *healthful* Plant-based Diet Index and the risk of newly-diagnosed chronic obstructive pulmonary disease in three cohorts of U.S. adults according-smoking status

	Health Professionals Follow-up Study (n=46,948)			Nurses' Health Study (n=73,592)			Nurses' Health Study II (n=85,515)			POOLED	
	No.	Person-Years	HR (95% CI)	No.	Person-Years	HR (95% CI)	No.	Person-Years	HR (95% CI)	No.	HR (95% CI)
**Never smokers**											
Multivariable-adjusted model 3*											
Quintile 1	9	90,919	1.00 (ref)	28	196,457	1.00 (ref)	49	321,827	1.00 (ref)	86	1.00 (ref)
Quintile 2	3	85,462	0.31 (0.08-1.16)	29	193,841	0.89 (0.53-1.51)	36	305,274	0.76 (0.49-1.18)	68	0.77 (0.55-1.06)
Quintile 3	8	83,778	0.76 (0.28-2.07)	39	187,310	1.17 (0.71-1.94)	41	299,203	0.93 (0.60-1.43)	88	0.99 (0.73-1.36)
Quintile 4	9	81,002	0.95 (0.35-2.56)	19	179,881	0.55 (0.30-1.01)	35	298,022	0.81 (0.51-1.29)	63	0.73 (0.51-1.03)
Quintile 5	7	76,047	0.80 (0.27-2.35)	36	177,918	0.93 (0.53-1.62)	26	271,977	0.68 (0.39-1.13)	69	0.78 (0.55-1.12)
P for trend			.76			.42			.22		.20
P value†											.73
I^2‡											0.0
**Former smokers**											
Multivariable-adjusted model 3*											
Quintile 1	80	78,055	1.00 (ref)	117	145,788	1.00 (ref)	38	89,958	1.00 (ref)	235	1.00 (ref)
Quintile 2	58	87,267	**0.64 (0.45-0.91)**	91	168,248	**0.60 (0.45-0.79)**	45	102,174	0.99 (0.64-1.54)	194	**0.68 (0.56-0.82)**
Quintile 3	59	92,904	**0.61 (0.43-0.86)**	82	174,897	**0.51 (0.38-0.69)**	50	118,987	0.97 (0.63-1.51)	191	**0.62 (0.51-0.76)**
Quintile 4	61	98,856	**0.63 (0.44-0.90)**	98	189,385	**0.55 (0.41-0.73)**	50	135,956	0.83 (0.53-1.32)	209	**0.62 (0.51-0.76)**
Quintile 5	38	96,450	**0.45 (0.30-0.69)**	77	197,630	**0.38 (0.28-0.53)**	28	143,882	**0.45 (0.27-0.77)**	143	**0.42 (0.33-0.52)**
P for trend			**<.001**			**<.001**			**.003**		**<.001**
P value†											.76
I^2‡											0.0
**Current smokers**											
Multivariable-adjusted model 3*											
Quintile 1	92	68,507	1.00 (ref)	164	57,543	1.00 (ref)	55	39,410	1.00 (ref)	311	1.00 (ref)
Quintile 2	90	62,585	1.00 (0.74-1.34)	140	51,838	**0.87 (0.69-1.09)**	41	36,468	0.88 (0.58-1.34)	271	0.91 (0.77-1.07)
Quintile 3	69	58,197	0.75 (0.54-1.04)	142	46,179	0.99 (0.78-1.26)	47	37,272	1.18 (0.78-1.80)	258	0.94 (0.79-1.12)
Quintile 4	68	58,750	0.75 (0.54-1.04)	122	41,742	**0.88 (0.68-1.13)**	31	36,218	0.82 (0.51-1.32)	221	**0.82 (0.68-0.99)**
Quintile 5	50	57,164	**0.59 (0.41-0.86)**	74	35,501	**0.59 (0.44-0.80)**	16	28,805	0.59 (0.32-1.08)	140	**0.59 (0.47-0.73)**
P for trend			**.001**			**.006**			.18		**<.001**
P value†											.69
I^2‡											0.0

A total of 701 cases occurred during 32 years of follow-up in the Health Professionals Follow-up Study (n=46,948). A total of 1,310 cases occurred during 34 years of follow-up in the Nurses' Health Study (n=73,592). A total of 594 cases occurred during 26 years of follow-up in the Nurses' Health Study II (n=85,515).

* Multivariable-adjusted model 3 includes age, pack-years of smoking (in ever smokers only; continuous), pack-years^2 of smoking (in ever smokers only; continuous), time since quitting smoking (in former smokers only; categorical), physical activity (metabolic equivalent task–hours/week, in quintiles), total caloric intake (continuous), US region (West, Midwest, South, or Northeast), race (white, or non-white), census tract median family income (continuous) and census tract median family home value (continuous).
† P value, test for between-studies heterogeneity.
‡ I^2, degree of heterogeneity between-studies expressed as a percent of total variance.

Table S6. Association between the *healthful* Plant-based Diet Index and *unhealthful* Plant-based Diet Index with the risk of newly-diagnosed chronic obstructive pulmonary disease using validated definition in three cohorts of U.S. adults

	Health Professionals Follow-up Study (n=46,948)			Nurses' Health Study (n=73,592)			Nurses' Health Study II (n=85,515)			POOLED	
	No.	Person-Years	HR (95% CI)	No.	Person-Years	HR (95% CI)	No.	Person-Years	HR (95% CI)	No.	HR (95% CI)
***healthful* Plant-based Diet Index**											
Multivariable-adjusted model 3*											
Quintile 1	52	103,733	1.00 (ref)	179	223,391	1.00 (ref)	137	457,424	1.00 (ref)	368	1.00 (ref)
Quintile 2	52	103,789	1.00 (0.68-1.48)	189	231,665	0.97 (0.79-1.20)	126	448,765	0.92 (0.72-1.18)	367	0.96 (0.82-1.11)
Quintile 3	38	102,324	0.72 (0.46-1.11)	190	227,088	1.04 (0.84-1.29)	134	459,792	1.01 (0.78-1.29)	362	0.98 (0.84-1.14)
Quintile 4	44	109,456	0.79 (0.51-1.21)	173	225,260	0.94 (0.75-1.18)	115	474,477	0.84 (0.64-1.09)	332	0.88 (0.75-1.03)
Quintile 5	23	102,321	**0.48 (0.28-0.81)**	126	232,316	**0.69 (0.54-0.89)**	71	449,279	**0.57 (0.41-0.78)**	220	**0.62 (0.51-0.74)**
P for trend			**.005**			**.01**			**.001**		**<.001**
P value†											.35
I^2‡											6.7
***unhealthful* Plant-based Diet Index**											
Multivariable-adjusted model 3*											
Quintile 1	47	105,305	1.00 (ref)	151	227,974	1.00 (ref)	106	453,468	1.00 (ref)	304	1.00 (ref)
Quintile 2	45	102,780	1.04 (0.69-1.58)	161	229,243	1.06 (0.85-1.32)	117	454,939	1.22 (0.93-1.59)	323	1.11 (0.95-1.30)
Quintile 3	46	103,613	1.07 (0.71-1.63)	187	229,707	1.22 (0.98-1.53)	129	458,221	**1.36 (1.04-1.78)**	362	**1.25 (1.07-1.46)**
Quintile 4	39	105,509	0.95 (0.61-1.47)	177	224,274	1.19 (0.95-1.50)	105	461,910	**1.14 (0.85-1.51)**	321	1.14 (0.99-1.32)
Quintile 5	32	104,415	0.87 (0.54-1.40)	181	228,523	1.24 (0.98-1.57)	126	461,198	**1.53 (1.15-2.03)**	339	**1.28 (1.08-1.51)**
P for trend			.53			**.04**			**.02**		**.009**
P value†											.20
I^2‡											37.7

Validated COPD cases were defined as participants who (1) previously self-reported a physician-diagnosed of chronic bronchitis or emphysema on the original main questionnaires, and (2) answered positively-physician-diagnosed chronic bronchitis, or physician-diagnosed emphysema, or physician-diagnosed COPD

on the supplemental COPD questionnaire, and (3) reported of a diagnostic test at diagnosis (pulmonary function testing, chest radiograph, or chest computed tomography).

A total of 209 cases occurred during 12 years of follow-up in the Health Professionals Follow-up Study (n=46,948). A total of 857 cases occurred during 16 years of follow-up in the Nurses' Health Study (n=73,592). A total of 583 cases occurred during 26 years of follow-up in the Nurses' Health Study II (n=85,515).

* Multivariable-adjusted model 3 includes age, smoking (never, former, current), pack -years of smoking (in ever smokers only; continuous), pack-years^2 of smoking (in ever smokers only; continuous), physical activity (metabolic equivalent task–hours/week, in quintiles), total caloric intake (continuous), US region (West, Midwest, South, or Northeast), race (white, or non-white), census tract median family income (continuous) and census tract median family home value (continuous).
† P value, test for between-studies heterogeneity
‡ I^2, degree of heterogeneity between-studies expressed as a percent of total variance.

Table S7. Time lagged associations (8 years and 12 years) between the *healthful* Plant-based Diet Index and *unhealthful* Plant-based Diet Index with the risk of newly-diagnosed chronic obstructive pulmonary disease in three cohorts of U.S. adults

	Health Professionals Follow-up Study (n=46,948)			Nurses' Health Study (n=73,592)			Nurses' Health Study II (n=85,515)			POOLED	
	No.	Person-Years	HR (95% CI)	No.	Person-Years	HR (95% CI)	No.	Person-Years	HR (95% CI)	No.	HR (95% CI)
						**Time lagged 8 years***					
***healthful* Plant-based Diet Index**											
Multivariable-adjusted model 3†											
Quintile 1	113	237,482	1.00 (ref)	194	430,770	1.00 (ref)	123	457,537	1.00 (ref)	430	1.00 (ref)
Quintile 2	95	235,315	0.79 (0.60-1.04)	136	444,623	**0.67 (0.53-0.83)**	111	448,787	0.91 (0.70-1.18)	342	**0.77 (0.66-0.89)**
Quintile 3	96	234,881	0.75 (0.57-1.00)	135	437,692	**0.66 (0.52-0.83)**	122	459,825	1.04 (0.80-1.35)	353	**0.79 (0.68-0.91)**
Quintile 4	99	238,608	0.79 (0.59-1.05)	123	441,737	**0.59 (0.46-0.75)**	99	474,506	0.82 (0.62-1.09)	321	**0.71 (0.61-0.83)**
Quintile 5	63	229,664	**0.53 (0.38-0.74)**	98	441,335	**0.47 (0.36-0.61)**	59	449,283	**0.54 (0.38-0.76)**	220	**0.50 (0.42-0.60)**
P for trend			**<.001**			**<.001**			**.001**		**<.001**
P value‡											.47
I^2§											0.0
***unhealthful* Plant-based Diet Index**											
Multivariable-adjusted model 3†											
Quintile 1	87	235,776	1.00 (ref)	119	444,459	1.00 (ref)	95	453,517	1.00 (ref)	301	1.00 (ref)
Quintile 2	99	235,991	1.17 (0.88-1.57)	118	446,783	0.99 (0.76-1.28)	100	454,972	1.16 (0.88-1.55)	317	1.09 (0.93-1.28)
Quintile 3	85	233,362	1.07 (0.78-1.45)	128	440,690	1.05 (0.81-1.36)	104	458,232	1.25 (0.93-1.67)	317	1.11 (0.95-1.31)
Quintile 4	103	236,895	1.29 (0.95-1.74)	146	434,061	1.23 (0.95-1.58)	99	461,979	1.20 (0.89-1.62)	348	**1.24 (1.05-1.46)**
Quintile 5	92	233,925	1.29 (0.94-1.77)	175	430,166	**1.47 (1.14-1.90)**	116	461,239	**1.60 (1.19-2.17)**	383	**1.46 (1.23-1.72)**
P for trend			.10			**<.001**			**.005**		**<.001**

P value‡											.60
I^2§											0.0
				**Time lagged 12 years¶**							
***healthful* Plant-based Diet Index**											
Multivariable-adjusted model 3†											
Quintile 1	76	237,482	1.00 (ref)	99	430,770	1.00 (ref)	104	457,537	1.00 (ref)	279	1.00 (ref)
Quintile 2	66	235,315	0.84 (0.60-1.17)	58	444,623	**0.59 (0.42-0.82)**	93	448,787	0.88 (0.66-1.17)	217	**0.77 (0.64-0.92)**
Quintile 3	64	234,881	0.77 (0.55-1.09)	59	437,692	**0.59 (0.42-0.83)**	105	459,825	1.04 (0.78-1.39)	228	**0.81 (0.67-0.97)**
Quintile 4	67	238,608	0.84 (0.59-1.18)	47	441,737	**0.47 (0.32-0.69)**	87	474,506	0.84 (0.61-1.14)	201	**0.71 (0.59-0.87)**
Quintile 5	47	229,664	**0.63 (0.43-0.93)**	45	441,335	**0.47 (0.32-0.70)**	55	449,283	**0.58 (0.41-0.83)**	147	**0.56 (0.45-0.70)**
P for trend			**.04**			**<.001**			**.01**		**<.001**
P value‡											.25
I^2§											27.6
***unhealthful* Plant-based Diet Index**											
Multivariable-adjusted model 3†											
Quintile 1	59	235,776	1.00 (ref)	51	444,459	1.00 (ref)	75	453,517	1.00 (ref)	185	1.00 (ref)
Quintile 2	71	235,991	1.23 (0.87-1.75)	52	446,783	1.01 (0.68-1.49)	87	454,972	1.26 (0.92-1.72)	210	1.18 (0.97-1.44)
Quintile 3	57	233,362	1.07 (0.73-1.55)	56	440,690	1.07 (0.72-1.58)	89	458,232	1.33 (0.96-1.82)	202	1.17 (0.95-1.44)
Quintile 4	72	236,895	1.33 (0.93-1.91)	64	434,061	1.24 (0.84-1.82)	87	461,979	1.31 (0.94-1.82)	223	**1.29 (1.05-1.59)**
Quintile 5	61	233,925	1.26 (0.85-1.85)	85	430,166	**1.64 (1.12-2.39)**	106	461,239	**1.83 (1.31-2.54)**	252	**1.58 (1.29-1.95)**
P for trend			.22			**.004**			**.001**		**<.001**
P value‡											.37
I^2§											0.0

* Time lagged analyses by omitting the cases from the initial eight years of follow-up: a total of 466 cases occurred during 32 years of follow-up in the Health Professionals Follow-up Study, a total of 686 cases occurred during 34 years of follow-up in the Nurses' Health Study and a total of 514 cases occurred during 26 years of follow-up in the Nurses' Health Study II.

† Multivariable-adjusted model 3 includes age, smoking (never, former, current), pack -years of smoking (in ever smokers only; continuous), pack-years^2 of smoking (in ever smokers only; continuous), physical activity (metabolic equivalent task–hours/week, in quintiles), total caloric intake (continuous), US region (West, Midwest, South, or Northeast), race (white, or non-white), census tract median family income (continuous) and census tract median family home value (continuous).

‡ P value, test for between-studies heterogeneity

§ I^2, degree of heterogeneity between-studies expressed as a percent of total variance.

¶ Time lagged analyses by omitting the cases from the initial twelve years of follow-up: a total of 320 cases occurred during 32 years of follow-up in the Health Professionals Follow-up Study, a total of 308 cases occurred during 34 years of follow-up in the Nurses' Health Study and a total of 444 cases occurred during 26 years of follow-up in the Nurses' Health Study II.

Table S8. Association between the *unhealthful* Plant-based Diet Index and the risk of newly-diagnosed chronic obstructive pulmonary disease in three cohorts of U.S. adults according-smoking status

	Health Professionals Follow-up Study (n=46,948)			Nurses' Health Study (n=73,592)			Nurses' Health Study II (n=85,515)			POOLED	
	No.	Person-Years	HR (95% CI)	No.	Person-Years	HR (95% CI)	No.	Person-Years	HR (95% CI)	No.	HR (95% CI)
**Never smokers**											
Multivariable-adjusted model 3*											
Quintile 1	5	75,646	1.00 (ref)	32	173,218	1.00 (ref)	32	263,567	1.00 (ref)	69	1.00 (ref)
Quintile 2	11	80,162	2.09 (0.71-6.12)	24	182,281	0.73 (0.43-1.24)	35	284,634	1.13 (0.70-1.84)	70	1.00 (0.71-1.41)
Quintile 3	7	82,116	1.48 (0.46-4.77)	29	188,265	0.90 (0.54-1.51)	41	299,138	1.32 (0.82-2.13)	77	1.13 (0.81-1.58)
Quintile 4	5	87,458	0.97 (0.27-3.51)	33	188,810	1.05 (0.63-1.75)	36	313,430	1.19 (0.72-1.97)	74	1.11 (0.78-1.56)
Quintile 5	8	91,826	1.66 (0.50-5.49)	33	202,833	1.02 (0.60-1.74)	43	335,535	1.44 (0.88-2.39)	84	1.26 (0.89-1.79)
P for trend			.95			.53			.18		.17
P value†											.84
I^2‡											0.0
**Former smokers**											
Multivariable-adjusted model 3*											
Quintile 1	53	100,337	1.00 (ref)	81	204,263	1.00 (ref)	43	151,893	1.00 (ref)	177	1.00 (ref)
Quintile 2	64	96,942	1.20 (0.83-1.74)	84	191,299	1.17 (0.86-1.60)	50	130,946	1.44 (0.96-2.19)	198	**1.25 (1.01-1.53)**
Quintile 3	56	91,590	1.09 (0.74-1.61)	101	174,814	**1.55 (1.15-2.09)**	46	118,766	1.52 (0.98-2.34)	203	**1.39 (1.13-1.71)**
Quintile 4	74	89,081	**1.53 (1.06-2.21)**	93	164,706	**1.55 (1.13-2.11)**	27	105,373	1.04 (0.63-1.74)	194	**1.43 (1.16-1.78)**
Quintile 5	49	75,583	1.16 (0.77-1.76)	106	140,865	**2.02 (1.48-2.76)**	45	83,979	**2.33 (1.47-3.71)**	200	**1.79 (1.43-2.23)**
P for trend			.20			**<.001**			**.009**		**<.001**
P value†											.17
I^2‡											45.8
**Current smokers**											
Multivariable-adjusted model 3*											
Quintile 1	78	59,791	1.00 (ref)	100	37,377	1.00 (ref)	32	33,625	1.00 (ref)	210	1.00 (ref)
Quintile 2	70	58,886	0.96 (0.69-1.33)	119	42,187	1.10 (0.84-1.44)	31	34,850	0.93 (0.56-1.54)	220	1.02 (0.84-1.24)
Quintile 3	66	59,655	0.93 (0.66-1.31)	132	47,878	1.12 (0.86-1.47)	42	35,647	1.15 (0.71-1.87)	240	1.06 (0.87-1.29)
Quintile 4	82	60,356	1.12 (0.81-1.56)	139	50,029	1.15 (0.88-1.51)	43	37,935	1.06 (0.65-1.73)	264	1.13 (0.93-1.37)
Quintile 5	73	66,515	0.99 (0.70-1.39)	152	55,332	1.21 (0.93-1.59)	42	36,115	1.11 (0.67-1.85)	267	1.12 (0.92-1.36)
P for trend			.73			.17			.57		.15
P value†											.85
I^2‡											0.0

A total of 701 cases occurred during 32 years of follow-up in the Health Professionals Follow-up Study (n=46,948). A total of 1,310 cases occurred during 34 years of follow-up in the Nurses' Health Study (n=73,592). A total of 594 cases occurred during 26 years of follow-up in the Nurses' Health Study II (n=85,515).

* Multivariable-adjusted model 3 includes age, pack-years of smoking (in ever smokers only; continuous), pack-years^2 of smoking (in ever smokers only;

continuous), time since quitting smoking (in former smokers only; categorical), physical activity (metabolic equivalent task–hours/week, in quintiles), total caloric intake (continuous), US region (West, Midwest, South, or Northeast), race (white, or non-white), census tract median family income (continuous) and census tract median family home value (continuous).

† P value, test for between-studies heterogeneity

‡ I^2, degree of heterogeneity between-studies expressed as a percent of total variance.

Figure S1. Flowchart of participants' selection in the Health Professional Follow-up Study, Nurses' Health Study, Nurses' Health Study II, and their respective study designs

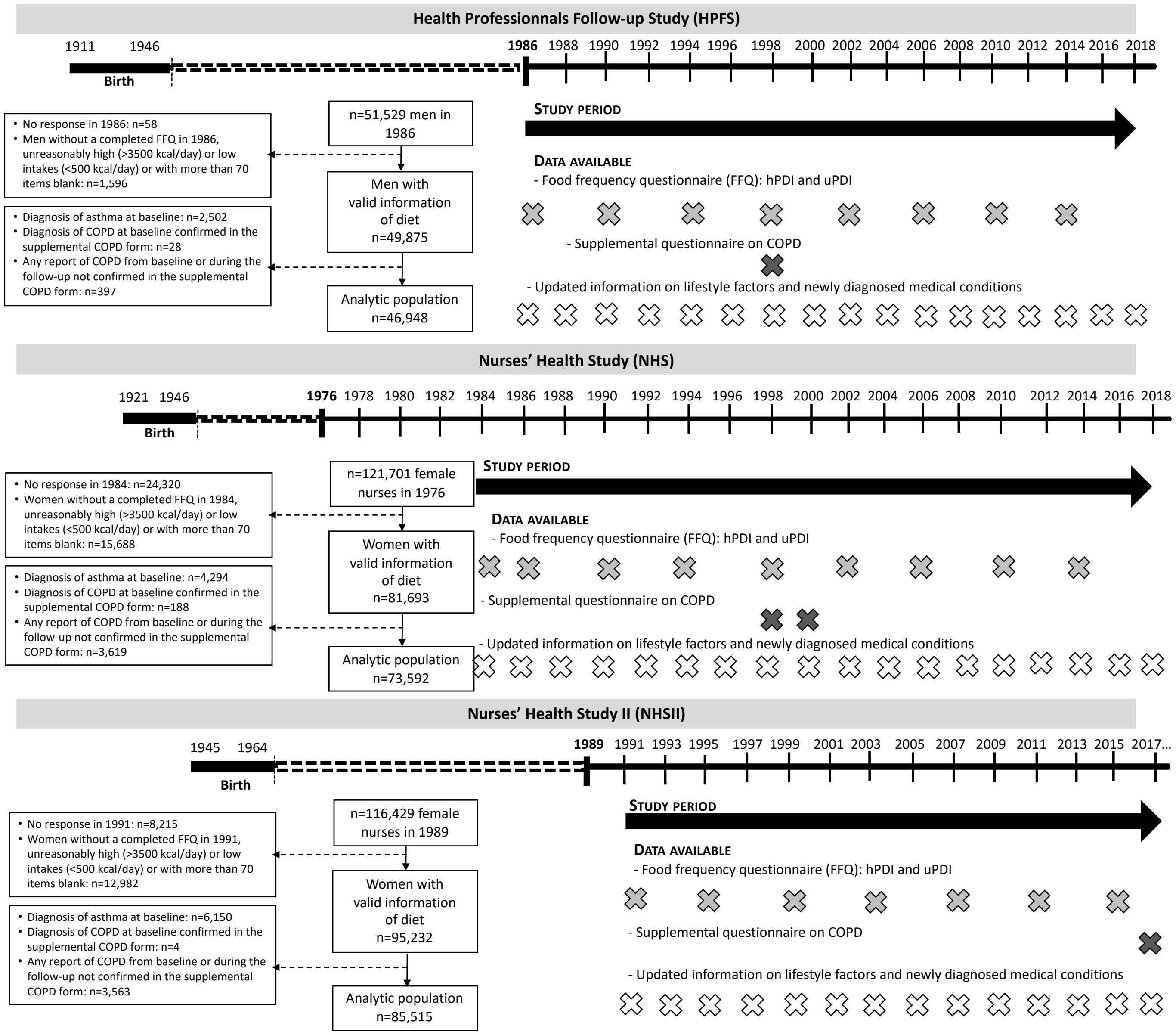

Supplement: Supplementary file 1 [file nutrients-15-00765-s001.zip › nutrients-2135032-supplementary.pdf]
